# In vitro efficacy of artemisinin-based treatments against SARS-CoV-2

**DOI:** 10.1038/s41598-021-93361-y

**Published:** 2021-07-16

**Authors:** Yuyong Zhou, Kerry Gilmore, Santseharay Ramirez, Eva Settels, Karen A. Gammeltoft, Long V. Pham, Ulrik Fahnøe, Shan Feng, Anna Offersgaard, Jakob Trimpert, Jens Bukh, Klaus Osterrieder, Judith M. Gottwein, Peter H. Seeberger

**Affiliations:** 1 Copenhagen Hepatitis C Program (CO-HEP), Department of Infectious Diseases, Copenhagen University Hospital-Hvidovre, Kettegård Alle 30, 2650 Hvidovre, Denmark; 2 CO-HEP, Department of Immunology and Microbiology, Faculty of Health and Medical Sciences, University of Copenhagen, Blegdamsvej 3B, 2200 Copenhagen, Denmark; 3 Max Planck Institute for Colloids and Interfaces, Am Mühlenberg 1, 14476 Potsdam, Germany; 4 Institute for Virology, Freie Universität Berlin, Robert von Ostertag-Str. 7-13, 14163 Berlin, Germany; 5 Department of Infectious Diseases and Public Health, Jockey Club College of Veterinary Medicine and Life Sciences, City University of Hong Kong, Kowloon Tong, Hong Kong; 6 Institute of Chemistry and Biochemistry, Freie Universität Berlin, Arnimallee 22, 14195 Berlin, Germany

**Keywords:** Viral infection, Drug discovery

## Abstract

Effective and affordable treatments for patients suffering from coronavirus disease 2019 (COVID-19), caused by severe acute respiratory syndrome coronavirus 2 (SARS-CoV-2), are needed. We report in vitro efficacy of *Artemisia annua* extracts as well as artemisinin, artesunate, and artemether against SARS-CoV-2. The latter two are approved active pharmaceutical ingredients of anti-malarial drugs. Concentration–response antiviral treatment assays, based on immunostaining of SARS-CoV-2 spike glycoprotein, revealed that treatment with all studied extracts and compounds inhibited SARS-CoV-2 infection of VeroE6 cells, human hepatoma Huh7.5 cells and human lung cancer A549-hACE2 cells, without obvious influence of the cell type on antiviral efficacy. In treatment assays, artesunate proved most potent (range of 50% effective concentrations (EC_50_) in different cell types: 7–12 µg/mL), followed by artemether (53–98 µg/mL), *A. annua* extracts (83–260 µg/mL) and artemisinin (151 to at least 208 µg/mL). The selectivity indices (SI), calculated based on treatment and cell viability assays, were mostly below 10 (range 2 to 54), suggesting a small therapeutic window. Time-of-addition experiments in A549-hACE2 cells revealed that artesunate targeted SARS-CoV-2 at the post-entry level. Peak plasma concentrations of artesunate exceeding EC_50_ values can be achieved. Clinical studies are required to further evaluate the utility of these compounds as COVID-19 treatment.

## Introduction

**Figure 1:**
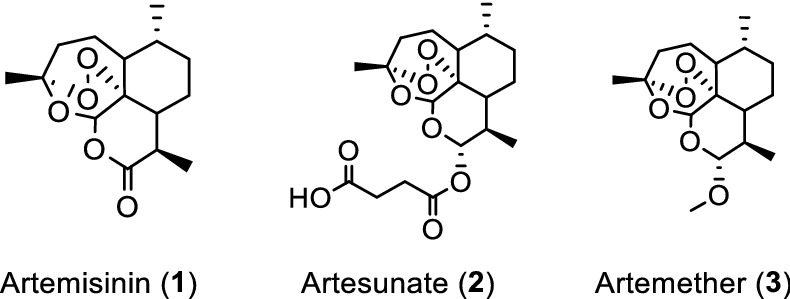
Artemisinin and related API derivatives artesunate and artemether.

**Figure 2:**
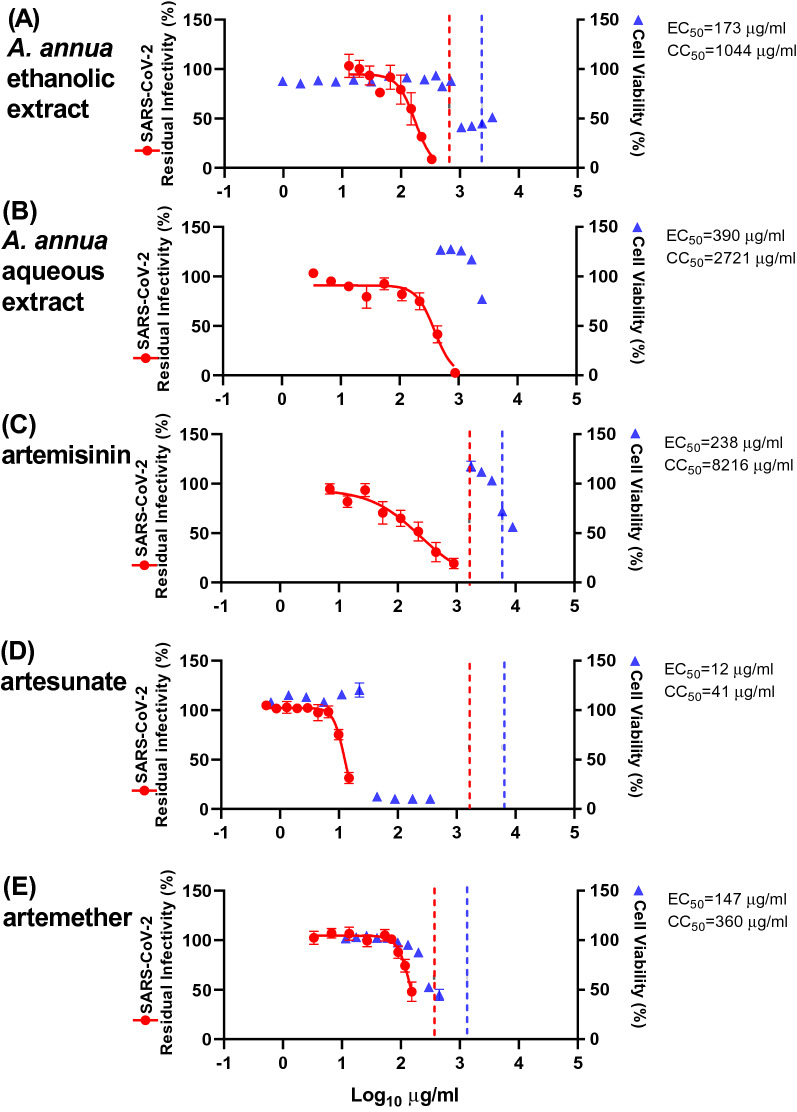
Pretreatment efficacy of extracts and compounds against SARS-CoV-2 in a concentration–response antiviral treatment assay in VeroE6 cells. VeroE6 cells seeded the previous day in 96-well plates were treated with the specified concentrations of extracts (**A**) *A. annua* ethanolic extract, and (**B**) *A. annua* aqueous extract, or compounds artemisinin (**C**), artesunate (**D**), and artemether (**E**) for 1.5 h prior to infection with SARS-CoV-2. After a 2-day incubation, infected cells were visualized by immunostaining for SARS-CoV-2 spike glycoprotein and counted automatically as described in “Materials and methods”. % residual infectivity for individual wells was calculated by relating counts of infected treated wells to the mean count of 14 infected nontreated control wells. Datapoints (red dots) are means of seven replicates with standard errors of the means (SEM) obtained in one representative experiment. Sigmoidal dose response curves (red lines) were fitted and EC_50_ values were calculated in GraphPad Prism as described in “Materials and methods”. % Cell viability and CC_50_ values were determined in replicate assays without infection with SARS-CoV-2 as described in “Materials and methods”. Datapoints (blue triangles) are means of 3 replicates with SEM obtained in one representative experiment. The dotted red/blue lines indicate the concentrations at which an antiviral effect (< 70% residual infectivity)/cytotoxic effect (< 90% cell viability) due to DMSO is expected according to Figure S6.

**Figure 3:**
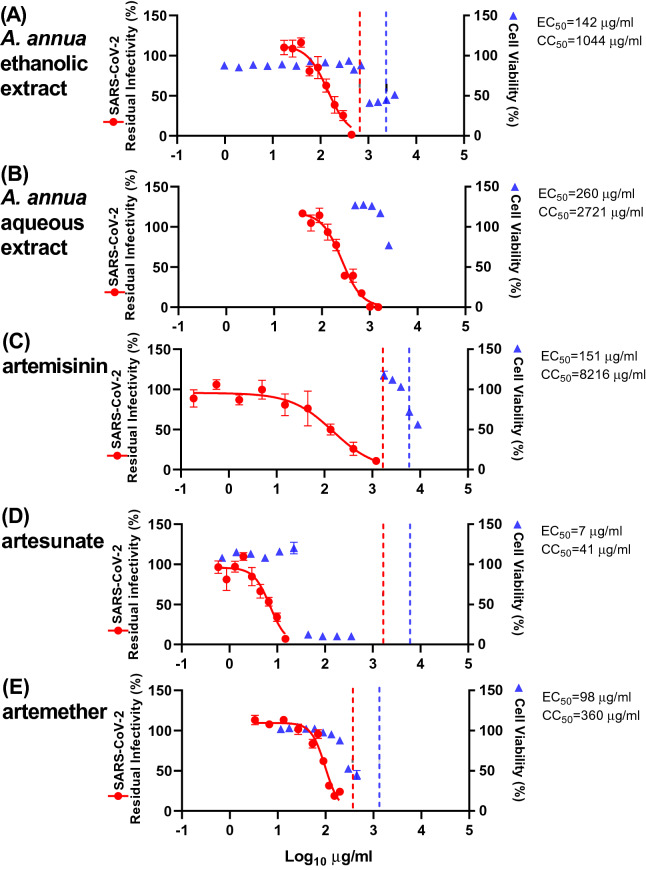
Treatment efficacy of extracts and compounds against SARS-CoV-2 in a concentration–response antiviral treatment assay in VeroE6 cells. VeroE6 cells seeded the previous day in 96-well plates were infected with SARS-CoV-2 and after 1 h incubation treated with the specified concentrations of extracts (**A**) *A. annua* ethanolic extract, and (**B**) *A. annua* aqueous extract or compounds artemisinin (**C**), artesunate (**D**), and artemether (**E**). After a 2-day incubation, infected cells were visualized by immunostaining for SARS-CoV-2 spike glycoprotein and counted automatically as described in “Materials and methods”. % residual infectivity for individual wells was calculated by relating counts of infected treated wells to the mean count of 14 infected nontreated control wells. Datapoints (red dots) are means of seven replicates with SEM obtained in one representative experiment. Sigmoidal dose response curves (red lines) were fitted and EC_50_ values were calculated in GraphPad Prism as described in “Materials and methods”. % Cell viability and CC_50_ values were determined in replicate assays without infection with SARS-CoV-2 as described in “Materials and methods”. Datapoints (blue triangles) are means of three replicates with SEM obtained in one representative experiment. The dotted red/blue lines indicate the concentrations at which an antiviral effect (< 70% residual infectivity)/cytotoxic effect (< 90% cell viability) due to DMSO is expected according to Figure S6.

The pandemic with severe acute respiratory syndrome coronavirus 2 (SARS-CoV-2)^1,2^ has until June 2021 worldwide been associated with over 3.9 million deaths from coronavirus disease 2019 (COVID-19)^3–5^. This febrile respiratory and systemic illness is highly contagious and in many cases life-threatening. Remdesivir is the only Food and Drug Administration (FDA) approved direct acting antiviral drug for treatment of COVID-19; however, its clinical efficacy has recently been challenged^6–8^. Thus, COVID-19 treatment remains largely supportive with an urgent need to identify additional antivirals against SARS-CoV-2. An attractive approach is repurposing drugs already licensed for other diseases. *A. annua* plants have been employed to treat malaria in Traditional Chinese Medicine, as well as in human trials^9,10^, and are used widely in many African countries, albeit against WHO recommendations. Artemisinin (Fig. 1, (**1)**), a sesquiterpene lactone with a peroxide moiety and one of many bioactive compounds present in *A. annua*, is the active ingredient to treat malaria infections^11,12^. The artemisinin derivatives artesunate (Fig. 1, (**2)**) and artemether (Fig. 1, (**3)**) exhibit improved pharmacokinetic properties and are the key active pharmaceutical ingredients (API) of WHO-recommended anti-malaria combination therapies used in millions of adults and children each year with few side effects^13^. *A. annua* extracts are active against different viruses, including SARS-CoV^14–16^. Therefore, we set out to determine whether *A. annua* extracts, as well as pure artemisinin, artesunate, and artemether are active against SARS-CoV-2 in vitro. To this aim we used different cell culture systems with permissiveness to SARS-CoV-2, the African green monkey kidney cell line VeroE6, the human hepatoma cell line Huh7.5 and the human lung cancer cell line A549 consitutively expressing human angiotensin converting enzyme 2 (ACE2) acting as SARS-CoV-2 entry receptor. Artemisinin-based drugs would be attractive repurposing candidates for treatment of COVID-19 considering their excellent safety profiles in humans, and since they are readily available for worldwide distribution at a relatively low cost.

## Results

### Extracts and compounds

*Artemisia annua* plants grown from a cultivated seed line in Kentucky, USA, were extracted using either absolute ethanol or distilled water at 50 °C for 200 min and analyzed, as described in “Materials and methods” and Supplementary Information (Figures S1 and S2). Solids were removed by filtration and the solvents were evaporated. The extracted materials were dissolved in dimethylsulfoxide (DMSO) (ethanol extract) or a DMSO:water mixture (3:1 for aqueous extract) and filtered (see supporting information for details). Artemisinin (Fig. 1, (**1**)) was synthesized and purified following a published procedure or purchased^17^, while artesunate (Fig. 1, (**2**)) and artemether (Fig. 1, (**3**)) were only obtained from commercial sources.

### Plaque-reduction assays in VeroE6 cells for in vitro proof-of-concept of the pretreatment efficacy of *A. annua* extracts and artemisinin

Initial experiments were carried out at FU Berlin, Germany. To initially screen whether extracts and pure artemisinin were active against SARS-CoV-2, their antiviral activity was tested by pretreating VeroE6 cells at different time points during 120 min with selected concentrations of the extracts or compounds prior to infection with the first European SARS-CoV-2 isolated in München (SARS-CoV-2/human/Germany/BavPat 1/2020). The virus-drug mixture was then removed and cells were overlaid with medium containing 1.3% carboxymethylcellulose to prevent virus release into the medium. DMSO was used as a negative control. Plaque numbers were determined either by indirect immunofluorescence using a mixture of antibodies to SARS-CoV N protein^18^ or by staining with crystal violet^19^. The addition of either ethanolic or aqueous *A. annua* extracts prior to virus addition resulted in reduced plaque formation in a concentration dependent manner, while artemisinin exhibited little antiviral activity (Supplemental Tables S1–S8).

### Efficacy of *A. annua* extracts in concentration–response antiviral treatment assays in VeroE6 cells

Concentration–response experiments were carried out at Copenhagen University Hospital-Hvidovre. In these experiments the Danish SARS-CoV-2 isolate SARS-CoV-2/human/Denmark/DK-AHH1/2020 was used employing a 96-well plate based concentration–response antiviral treatment assay, allowing for multiple replicates per concentration, as described in “Materials and methods” and Supplementary Information (Figures S3 and S4)^20,21^. Seven replicates were measured at each concentration and a range of concentrations was evaluated to increase data accuracy when compared to the plaque-reduction assay, which was carried out in duplicates. Extracts or compounds were added to VeroE6 cells either 1.5 h prior to (pretreatment (pt)) or 1 h post infection (treatment (t)), respectively, followed by a 2-day incubation of virus with extracts or compounds. Both protocols yielded similar results, with slightly lower median effective concentration (EC_50_) values observed for treatment assays.

The ethanolic extract exhibited an EC_50_ of 173 µg/mL (pt) and 142 µg/mL (t) (Figs. 2, 3 and Table 1), while the aqueous extract was slightly less potent with EC_50_ being 390 µg/mL (pt) and 260 µg/mL (t) (Figs. 2, 3 and Table 1). With both extracts, almost complete virus inhibition was achieved at high concentrations: for the *A. annua* ethanolic extract at 333 µg/mL (pt) and 444 µg/mL (t) and the *A. annua* aqueous extract at 875 µg/mL (pt) and 1009 µg/mL (t) (Figs. 2 and 3). The highest evaluated concentrations used in our assays were informed by the cytotoxicity of the extracts or compounds, as only concentrations resulting in cell viability greater than 85% were evaluated (Figs. 2, 3, S5 and Table 1). Cell viability assays revealed median cytotoxic concentrations (CC_50_) of 1044 µg/mL (*A. annua* ethanolic extract) and 2721 µg/mL (*A. annua* aqueous extract) (Figs. 2, 3, S5 and Table 1). Selectivity indexes (SI) were determined by dividing CC_50_ by EC_50_ and revealed similar results for the *A. annua* ethanolic extract (6 (pt) and 7 (t)) and the *A. annua* aqueous extract (7 (pt) and 10 (t)) (Table 1).

**Table 1:** Efficacy of extracts and compounds in vitro.

	EC50 (μg/ml)^a^		CC_50_ (μg/ml)^b^	SI^c^
	Pretreatment assay	Treatment assay		
**VeroE6 cells**				
Extract				
*A. annua* ethanolic extract	173	142	1044	6/7
*A. annua* aqueous extract	390	260	2721	7/10
Compound				
Artemisinin	238	151	8216	35/54
Artesunate	12	7	41	3/6
Artemether	147	98	360	2/4
**Huh7.5 cells**				
Extract				
*A. annua* ethanolic extract	118		483	4
Compound				
Artemisinin	> 208		5066	< 24
Artesunate	11		93	8
Artemether	64		127	2
**A549-hACE2 cells**				
Extract				
*A. annua* ethanolic extract	83		506	6
Compound				
Artemisinin	168		1527	9
Artesunate	12		27	2
Artemether	53		380	7

^a^EC_50_, median effective concentration (µg/mL) was determined in VeroE6 cells in pretreatment or treatment antiviral assays or in Huh7.5 cells and A549-hACE2 cells in treatment antiviral assays as described in Material and Methods. For artemisinin in Huh7.5 cells, < 50% inhibition was observed at the highest non-cytotoxic concentration where cell viability was > 90% of that of non-treated control cultures.

^b^CC_50_, median cytotoxic concentration (µg/mL) was determined as described in Material and Methods.

^c^SI, selectivity index, was determined as CC_50_ divided by EC_50_ based on results in pretreatment/treatment antiviral assays in VeroE6 cells or based on results in treatment antiviral assays in Huh 7.5 cells and A549-hACE2 cells.

The ethanolic extract was diluted with DMSO that by itself caused reduction of cell viability to < 90% when used at a 1:28 dilution, but not at dilutions ≥ 1:42 (Figure  S6). Thus, the cytotoxicity observed when using the extract at relatively high concentrations was most likely not caused by DMSO (Figs. 2 and 3). DMSO at dilutions > 1:152, including the dilutions used in antiviral assays, did not have antiviral effects, defined as reduction of residual infectivity to < 70% (Figure S6). Thus, the observed antiviral effect of the tested extract was most likely not caused by DMSO.

### Efficacy of artemisinin and its derivatives in concentration–response antiviral treatment assays in VeroE6 cells

*Artemisia annua* plants contain, in addition to many other bioactive compounds, artemisinin that is responsible for the potent anti-malarial activities of *A. annua*. To investigate whether artemisinin is the active component responsible for the antiviral activities of the plant extracts described above, the pure compound and synthetic derivatives were tested in pretreatment and treatment assays. Artemisinin was found to be active in SARS-CoV-2 assays with EC_50_ 238 µg/mL (pt) and 151 µg/mL (t) (Figs. 2, 3, and Table 1). Close to complete virus inhibition was achieved in both assays at the highest concentration evaluated in the assays, 893 (pt) and 1208 µg/mL (t). The SI for artemisinin is relatively high, 35 (pt) and 54 (t), based on a CC_50_ of 8,216 µg/mL (Figs. 2, 3, S5, and Table 1). The observed cytotoxicity of artemisinin appeared to be at least partially caused by DMSO, as cytotoxicity was only observed at drug dilutions where DMSO was found to reduce cell viability (Figs. 2, 3, and S6). The antiviral effects observed when using artemisinin at relatively high concentrations were most likely not due to the diluent DMSO (Figs. 2, 3, and S6).

The synthetic artemisinin derivative artesunate, the API of WHO-recommended first-line malaria therapies with improved pharmacokinetic properties, showed the highest potency of all compounds tested, with EC_50_ being 12 µg/mL (pt) and 7 µg/mL (t) (Figs. 2, 3 and Table 1). In the treatment assay, close to complete virus inhibition was achieved at the highest evaluated concentration (15 µg/mL), as determined by cytotoxicity data, compared to 69% inhibition at this concentration in the pretreatment assay. Higher artesunate concentrations were not used considering its cytotoxicity in this assay (CC_50_: 41 µg/mL) (Figs. 2, 3, S5, and Table 1). SI of 3 (pt) and 6 (t) were calculated (Table 1). The cytotoxicity and the antiviral effects observed when using artesunate at relatively high concentrations were most likely not due to the diluent DMSO (Figs. 2, 3, and S6).

Artemether, another artemisinin-derivative that is used globally as the active ingredient in malaria medications, showed intermediate potency with EC_50_ 147 µg/mL (pt) and 98 µg/mL (t) (Figs. 2, 3, and Table 1). In the treatment assay, close to complete virus inhibition was achieved at the highest concentrations (≥ 153 µg/mL), as determined by cytotoxicity data, compared to 52% inhibition at this concentration in the pretreatment assay. Considering artemether´s cytotoxicity (CC_50_ of 360 µg/mL), SI of 2 (pt) and 4 (t) were calculated (Figs. 2, 3, S5, and Table 1). The cytotoxicity observed when using artemether at relatively high concentrations was most likely not due to the diluent DMSO (Figs. 2, 3, and S6).

### Efficacy of artemisinin-based treatment in concentration–response antiviral treatment assays using Huh7.5 cells

**Figure 4:**
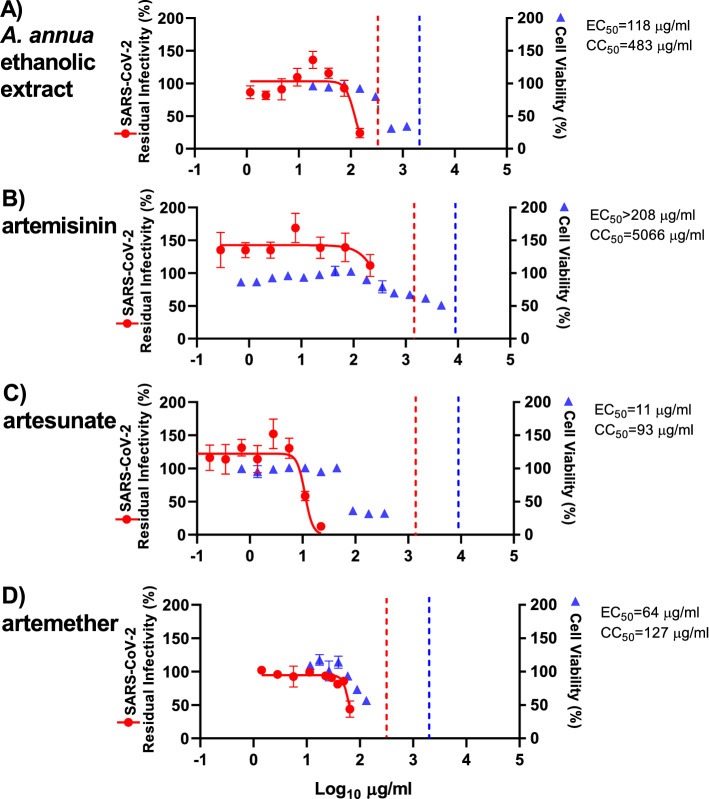
Treatment efficacy of *A. annua* extract and compounds against SARS-CoV-2 in a concentration–response antiviral treatment assay in Huh7.5 cells. Huh7.5 cells seeded the previous day in 96-well plates were infected with SARS-CoV-2 and directly treated with the specified concentrations of *A. annua* ethanolic extract (**A**) or compounds artemisinin (**B**), artesunate (**C**), and artemether (**D**). After a 3-day incubation, infected cells were visualized by immunostaining for SARS-CoV-2 spike glycoprotein and counted automatically as described in “Materials and methods”. % residual infectivity for individual wells was calculated by relating counts of infected treated wells to the mean count of 14 infected nontreated control wells. Datapoints (red dots) are means of seven replicates with SEM obtained in one representative experiment. Sigmoidal dose response curves (red lines) were fitted and EC_50_ values were calculated in GraphPad Prism as described in “Materials and methods”. % Cell viability and CC_50_ values were determined in replicate assays without infection with SARS-CoV-2 as described in “Materials and methods”. Datapoints (blue triangles) are means of 3 replicates with SEM obtained in one representative experiment. The dotted red/blue lines indicate the concentrations at which an antiviral effect (< 70% residual infectivity)/cytotoxic effect (< 90% cell viability) due to DMSO is expected according to Figure S8.

The observed antiviral activity in these assays is affected by the ability of the pure compounds, and the compounds contained in the extracts, to enter the cells as well as their rates of metabolism within the cells. To exclude major differences in potency of extracts and compounds in human cells, treatment assays were also carried out in human hepatoma Huh7.5 cells, adding extracts or compounds to the cells immediately post infection. Overall, the ethanolic *A. annua* extract, artemisinin, artesunate, and artemether showed similar efficacy in Huh7.5 compared to VeroE6 cells. Artesunate (EC_50_: 11 µg/mL) was again found to be the most potent compound with close to complete virus inhibition at 22 µg/mL and an SI of 8 as determined by a CC_50_ of 93 µg/mL (Figs. 4, S7 and Table 1). Artemether, (EC_50_: 64 µg/mL) with 56% virus inhibition at the highest evaluated concentration (65 µg/mL), had an SI of 2, based on CC_50_ of 127 µg/mL (Figs. 4, S7 and Table 1). In Huh7.5 cells, the EC_50_ for the ethanolic *A. annua* extract was 118 µg/mL, with 76% virus inhibition at the highest evaluated concentration (150 µg/mL), as determined by cytotoxicity data; the CC_50_ was 483 µg/mL and the SI was 4 (Figs. 4, S7 and Table 1). Artemisinin showed no significant virus inhibition at the highest evaluated concentration (208 µg/mL) and an SI < 24, based on a CC_50_ of 5066 µg/mL (Figs. 4, S7 and Table 1).

In Huh7.5 cells, DMSO caused reduction of cell viability to < 90% when used at a 1:28 dilution, but not at dilutions ≥ 1:56 (Figure  S8). Thus, the cytotoxicity observed when using the ethanolic extract or the pure compounds at relatively high concentrations was most likely not caused by DMSO (Figure 4). DMSO at dilutions > 1:179 including dilutions used in antiviral assays did not have any antiviral effects (Figure S8). Thus, the observed antiviral effect of the ethanolic *A. annua* extract and the pure compounds was most likely not caused by DMSO.

### Efficacy of artemisinin-based treatment in concentration–response antiviral treatment assays using A549-hACE2 cells

**Figure 5:**
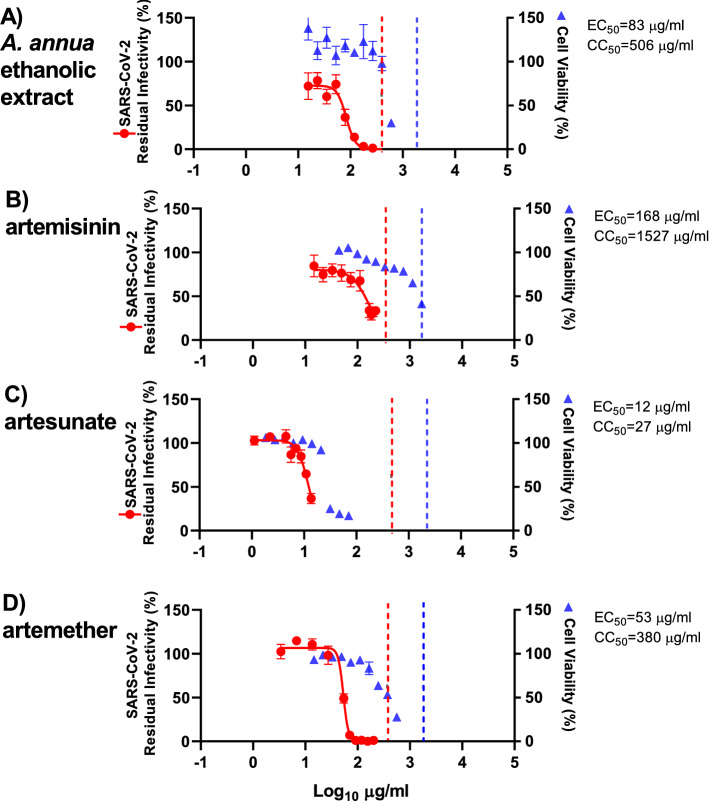
Treatment efficacy of *A. annua* extract and compounds against SARS-CoV-2 in a concentration–response antiviral treatment assay in A549-hACE2 cells. A549-hACE2 cells seeded the previous day in 96-well plates were infected with SARS-CoV-2 and directly treated with the specified concentrations of *A. annua* ethanolic extract (**A**) or compounds artemisinin (**B**), artesunate (**C**), and artemether (**D**). After a 2-day incubation, infected cells were visualized by immunostaining for SARS-CoV-2 spike glycoprotein and counted automatically as described in “Materials and methods”. % residual infectivity for individual wells was calculated by relating counts of infected treated wells to the mean count of 14 infected nontreated control wells. Datapoints (red dots) are means of seven replicates with SEM obtained in one representative experiment. Sigmoidal dose response curves (red lines) were fitted and EC_50_ values were calculated in GraphPad Prism as described in “Materials and methods”. % Cell viability and CC_50_ values were determined in replicate assays without infection with SARS-CoV-2 as described in “Materials and methods”. Datapoints (blue triangles) are means of 3 replicates with SEM obtained in one representative experiment. The dotted red/blue lines indicate the concentrations at which an antiviral effect (< 70% residual infectivity)/cytotoxic effect (< 90% cell viability) due to DMSO is expected according to Figure S10.

To verify our results in another physiologically relevant cell culture model for antiviral studies on SARS-CoV-2, we carried out treatment assays in A549 human lung cancer cells stably expressing the human SARS-CoV-2 entry receptor ACE2 (A549-hACE2 cells). In these assays, the ethanolic *A. annua* extract, artemisinin, artesunate, and artemether showed similar efficacy as in VeroE6 and Huh7.5 cells. Artesunate (EC_50_: 12 µg/mL) was again the most potent compound. However high cytotoxicity in A549-hACE2 cells (SI of 2 determined by CC_50_ of 27 µg/mL) precluded testing of significantly higher concentrations resulting in 63% virus inhibition at the highest evaluated concentration (14 µg/mL) (Figs. 5, S9, and Table 1). Artemether, (EC_50_: 53 µg/mL) with close to complete virus inhibition at 92 µg/mL, had an SI of 7, based on CC_50_ of 380 µg/mL (Figs. 5, S9, and Table 1). The EC_50_ for the ethanolic *A. annua* extract was 83 µg/mL with close to complete virus inhibition at 178 µg/mL; the CC_50_ was 506 µg/mL and the SI was 6 (Figs. 5, S9, and Table 1). For Artemisinin the EC_50_ was 168 µg/mL; however relatively high cytotoxicity in A549-hACE2 cells (SI of 9 determined by CC_50_ of 1527 µg/mL) precluded testing of higher concentrations (Figs. 5, S9, and Table 1).

In A549-hACE2 cells, DMSO caused reduction of cell viability to < 90% when used at a 1:32 dilution, but not at dilutions ≥ 1:64 (Figure  S10). Thus, the cytotoxicity observed when using the ethanolic extract or the pure compounds at relatively high concentrations was most likely not caused by DMSO (Figure. 5). DMSO at dilutions > 1:141 including dilutions used in antiviral assays did not have any antiviral effects (Figure S10). Thus, the observed antiviral effect of the ethanolic *A. annua* extract and the pure compounds was most likely not caused by DMSO.

### Artesunate blocked SARS-CoV-2 infection at the post-entry level

**Figure 6:**
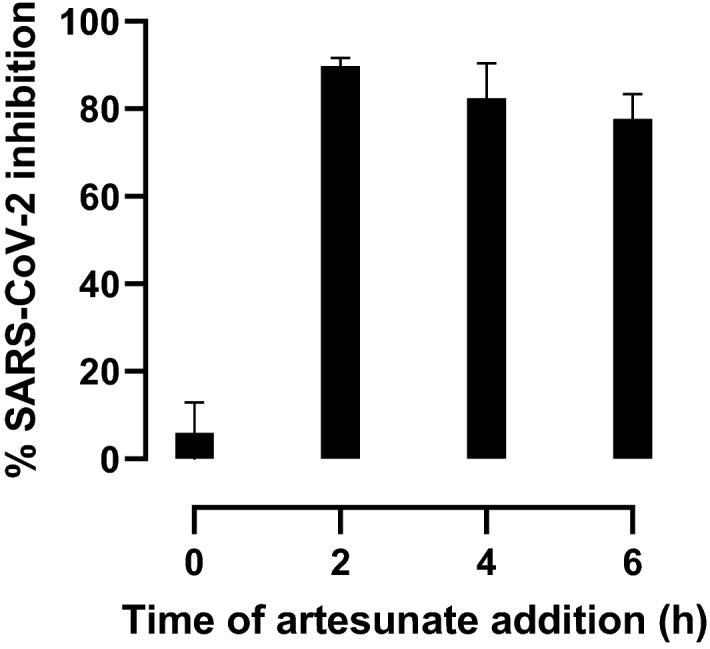
Time-of-artesunate-addition assay. A549-hACE2 cells were infected with SARS-CoV-2 for 2 h. Artesunate at 14 µg/mL was added at different time points after viral inoculation: 0 h, addition at the time of viral inoculation with presence of the drug during the 2 h viral infection phase; 2 h, addition 2 h post inoculation, immediately after the 2 h viral infection phase; 4 h and 6 h, addition 4 h and 6 h post inoculation, respectively. After a 2-day incubation, infected cells were visualized by immunostaining for SARS-CoV-2 spike glycoprotein and counted automatically as described in “Materials and methods”. % inhibition was calculated as (100%—% residual infectivity). % residual infectivity for individual wells was calculated by relating counts of infected treated wells to the mean count of 12 infected nontreated control wells. Datapoints (columns) are means of six replicates with SEM.

To gain insight into the mechanism of action of artenusate, the most potent compound investigated in this study, we carried out a time-of-addition assay in A549-hACE2 cells. We observed that addition of artesunate at the time of viral inoculation (0 h post inoculation) in the presence of the drug during the two hour viral infection phase did not result in inhibition of SARS-CoV-2 infection (Figs. 6 and S11). In contrast, when artesunate was added to cells at different timepoints following the 2 h viral infection phase (2 h, 4 h, and 6 h post inoculation), at least 80% inhibition of viral infection was observed at all times. Apparently, artesunate targets SARS-CoV-2 at the post-entry level.

## Discussion

Here, we demonstrate the in vitro efficacy of artemisinin-based treatments against SARS-CoV-2. Initially, several *A. annua* extracts, as well as artemisinin, were screened for antiviral activity using a plaque-reduction assay in VeroE6 cells in a pretreatment setting using a German SARS-CoV-2 strain from Munich. Based on these findings, three *A. annua* extracts and pure, synthetic artemisinin, artesunate, and artemether were studied in detail to establish concentration–response curves for extracts and compounds for pretreatment and treatment settings using a Danish SARS-CoV-2 strain from Copenhagen in VeroE6 cells. Finally, efficacy of an *A. annua* extract as well as artemisinin, artesunate, and artemether against the Danish SARS-CoV-2 strain was confirmed in Huh7.5 and A549-hACE2 cells.

Concentration–response antiviral treatment assays facilitated testing of drug concentrations in multiple replicates resulting in accurate EC_50_ values. In VeroE6 cells, the EC_50_ values in the pretreatment setting were slightly higher than EC_50_ values determined in the treatment setting. Pre-incubation may have a negative impact on the stability of the extracts and pure compounds, especially if they target SARS-CoV-2 at a late step of the viral life cycle, as demonstrated here for artesunate and previously for arteannuin B, another artemisinin derivative^22^. Generally, EC_50_ values depend on the specific assay employed. While the type of assay we used with a single treatment and subsequent incubation of virus and drug is state of the art for antiviral efficacy measurements, assay modifications, such as repeated administration of treatment, might result in slightly different EC_50_ values. Since the active antiviral substance may be an artemisinin metabolite, such that the artemisinin derivatives and extracts can be considered prodrugs, we used the human hepatoma cell line Huh7.5 and a human lung carcinoma cell line A549 constitutively expressing the SARS-CoV-2 entry receptor human ACE2 (A549-hACE2 cells) to confirm the EC_50_ determined in VeroE6 cells. Thus, this study is the first to show activity of artemisinin-based treatment against SARS-CoV-2 in human cell lines, including lung cells.

While *A. annua* extracts have been considered “natural combination therapies” as they contain several bioactive compounds^23^, the WHO discourages the use of non-pharmaceutical forms of artemisinin as a therapeutic option for malaria due to lack of standardization with its sourcing and preparation, implying risks of suboptimal efficacy and resistance development^24^. In this context, it is important to note that the extracts used in this study were prepared from plants grown under optimized and standardized conditions, in a manner where concentrations of the extracted material are reproducible (see supporting information for details).

While compiling data for this study, Cao et al. reported efficacy of artemisinin derivatives against a SARS-CoV-2 isolate from Wuhan in VeroE6 cells^22^. While extracts were not studied, the efficacy of artesunate was similar with EC_50_ of 13 µM compared to 18 µM in our VeroE6 treatment assay. EC_50_ for artemether and artemisinin were fourfold and eightfold higher in our VeroE6 treatment assay compared to values reported by Cao et al*.* This difference might be due to the nature of the assay employed. The assay used by Cao et al*.* was based on viral RNA determinations, the viral inoculum was removed post infection/prior to treatment and perhaps most importantly, the assay was terminated 24 h post infection, which is expected to result in comparatively lower EC_50_ for compounds with a limited capacity to control the virus. Future studies might address why artemisinin and artemether showed lower potency than artesunate. Finally, in our study we confirm efficacy of artemisinin-based treatment for two European SARS-CoV-2 strains from Germany and Denmark, which are more closely related to the majority of SARS-CoV-2 strains circulating worldwide than the Wuhan strain.

Given our findings for artesunate and findings by Cao et al. for arteannuin B, artemisinin-based treatment is likely to target SARS-CoV-2 at the post-entry level. Future studies will be required to confirm this observation for different artemisinin derivatives and plant extracts. While an antiviral effect of artemisinin, its derivatives and plant extracts has been reported for several viruses, the precise mechanisms of action remain to be elucidated^25^. Artesunate, the API in FDA approved malaria treatments, showed the highest potency against SARS-CoV-2 among the extracts and pure compounds tested in VeroE6, Huh7.5, and A549-hACE2 cells, followed by artemether, *A. annua* extracts, and artemisinin. SI of the tested extracts and compounds were relatively low (mostly < 10), suggesting a relatively small therapeutic window. It should be noted that certain drugs such as digoxin with SI values as low as 2 are used successfully in the clinic^26^. Among the tested extracts and pure compounds, only artesunate showed EC_50_ values in the range of clinically achievable plasma and tissue concentrations. When the typically used doses of 2 to 2.4 mg/kg intravenously were administered, reported peak plasma concentrations (Cmax) were between 19.4 and 29.7 µg/mL in patients^27^. Based on these observations and our treatment data in VeroE6, Huh7.5, and A549-ACE2 cells, the calculated Cmax/EC_50_ values are between 1.6 and 4.2. In animal studies following administration of a single dose of artesunate, tissue concentrations including lung, kidney, intestine, and spleen concentrations were several-fold higher than plasma concentrations^28^. In contrast, following administration of artemether, Cmax values between 6 and 190 ng/mL were reported, which is two to several orders of magnitude below determined EC_50_ values. Following administration of artemisinin and *A. annua* teas, Cmax values between 240  and 776 ng/mL were reported, which is two to three orders of magnitude below determined EC_50_ values. Plasma and tissue concentrations that can be achieved with standardized *A. annua* extracts with high artemisinin content used in this study still have to be determined. In vivo, immunomodulatory effects of artemisinin-based treatments have been reported for this class of drugs^29^. Such effects that may involve cytokine signaling cannot be monitored in in vitro assays performed here and are currently being studied in ongoing phase 1 and 2 clinical trials.

## Materials and methods

### Preparation of *A. annua* extracts

Solvent (250 mL ethanol or distilled water) was heated to 50 °C in an Erlenmeyer flask. Dried plant material (50 g for ethanol, 25 g for water) was added to the solvent and stirred for 200 min. The mixture was filtered and solid material washed with fresh ethanol or water. The solvent was removed by rotary evaporation and solid material stored at − 30 °C prior to sample preparation. Dried extract was warmed to room temperature. The required sample mass was removed using a spatula. DMSO (3 mL, ethanol extracts) or DMSO:water (3:1, 8 mL water extract) was added and the mixture was heated (40 °C) to ensure solvation. The solution was filtered using a syringe filter and stored in a snap-close vial. Further details are provided in Supplementary Information.

### Qualitative HPLC analysis of extracts for dihydroartemisinic acid and artemisinin

Ethanolic *A. annua* extracts were analyzed using an Agilent 1260 Series system composed of an auto sampler, a binary pump and a column oven coupled to an evaporative light scattering detector (Agilent Infinity II ELSD) and mass spectrometry detector (Agilent). The extract in acetonitrile was filtered using 0.45 µm cellulose syringe filters prior to analysis. Using acetonitrile/water + 0.1 vol% formic acid (80/20 v/v) at 1 mL/min the sample was passed through a Synergi C18 (250 × 4.6 mm, 4.6 µm) column (Phenomenex, Germany). An example for the obtained chromatogram is shown in Figure S2.

### Compounds

Artemisinin was either synthesized and purified as described in Supplementary Information or purchased (Sigma, Saint Louis, Missouri, USA). Artesunate was purchased (Selleckchem, Houston, Texas, USA or TCI, Eschborn, Germany). Artemether was purchased (Selleckchem, Houston, USA). Compounds were dissolved in DMSO and frozen. Details on sample prepration and application of compounds are provided in Supplementary Information.

### Cell culture

At FU Berlin, African green monkey kidney VeroE6 cells (ATCC CRL-1586) were maintained at 37 °C with 5% CO_2_ in Minimum Essential Medium (MEM; PAN Biotech, Aidenbach, Germany) supplemented with 10% fetal bovine serum (FBS) (PAN Biotech), 100 IU/mL penicillin G and 100 µg/mL streptomycin (Carl Roth, Karlsruhe, Germany).

At CO-HEP, African green monkey kidney VeroE6 cells (kind gift from Prof. Jean Dubuisson) as well as human hepatoma Huh7.5 cells^30^ were maintained at 37 °C with 5% CO_2_ in Dulbecco’s Modified Medium (DMEM) (Invitrogen, Paisley, UK) containing 10% heat inactivated FBS (Sigma, Saint Louis, Missouri, USA) and 100 U/mL penicillin + 100 µg/mL streptomycin (Gibco/Invitrogen Corporation, Carlsbad, California, USA). A549-hACE2 cells (Invivogen, Toulouse, France) were maintained at 37 °C with 5% CO_2_ in Dulbecco's Modified Eagle Medium: Nutrient Mixture F-12 (Gibco, Paisley, UK) containing 10% heat inactivated FBS (Sigma, Saint Louis, Missouri, USA), 100 U/mL penicillin + 100 μg/mL streptomycin (Gibco/Invitrogen Corporation, Carlsbad, California, USA) and 0.5 µg/mL puromycin (Invivogen, Toulouse, France). Cells were sub-cultured every 2–3 days using trypsin (Sigma, Saint Louis, Missouri, USA) to maintain a sub-confluent cell layer.

### Virus isolates

The SARS-CoV-2 isolate SARS-CoV-2/human/Germany/BavPat 1/2020 was provided by Dr. Daniela Niemeyer and Prof. Christian Drosten (Charité, Berlin, Germany) and obtained from an outbreak in Munich, Germany, in February 2020 .

The SARS-CoV-2/human/Denmark/DK-AHH1/2020 virus for cell culture studies was obtained following inoculation of VeroE6 cells with patient swab sample, virus propagation in VeroE6 cells and generation of a sequence confirmed 2nd viral passage stock with an infectivity titer of 5.5 log TCID50/mL as described in Ramirez et al.^20^.

### Plaque reduction antiviral assay

Antiviral activity of artemisinin derivatives was evaluated on VeroE6 cells grown overnight in 12-well plates (Sarstedt) at a density of approximately 5 × 10^5^ cells/well. Cells were incubated in the presence of ten-fold serial dilutions of the compounds for 15 min, 30 min, 60 min or 120 min, before the virus was added at a concentration of approximately 200 plaque-forming-units (PFU) per well for 120 min. The virus-drug mixture was removed, and cells were overlaid with MEM-FBS containing 1.3% carboxymethylcellulose to prevent virus release into the medium. DMSO in cell culture medium at a 1:100 dilution (the highest concentration relative to the preparations of extracts/compounds) was used as a negative control, and virus plaque numbers were determined by manual counting of plaques following indirect immunofluorescence (IF) using a mixture of antibodies to SARS-CoV N protein^18^ or following staining with crystal violet^19^. For IF, cells were fixed with 4% formalin and permeabilized with 0.25% Triton X-100. Unspecific binding was blocked with 1% FBS in phosphate buffered saline (PBS) containing 0.25% Triton X-100 (PBS-T) at room temperature for 30 min. Cells were incubated with the anti-N monoclonal antibodies (1:25 dilution in PBS-T) for 45 min, followed by incubation with secondary antibody (Alexa 488-labeled goat anti-mouse at a 1:500 dilution; Thermo Fisher). In each assay, each concentration was tested in one replicate culture; five infected and DMSO control treated cultures were included in each assay. Plaque counts recorded in each infected treated culture were related to the average count of the five control cultures to calculate the number of plaques as percent relative to the control. Two independent assays were carried out. Datapoints are means of two replicate cultures from the two independent assays with error bars reflecting the standard deviations (SD) (Table S1-S8). Selected concentrations were only tested in one of the assays and for these datapoints are based on single replicates. The multiplicity of infection (MOI) for infection was chosen aiming at on average 150–250 plaques per culture.

### Concentration–response pretreatment and treatment antiviral assay in VeroE6 cells

96-well based antiviral assays in VeroE6 cells were developed based on assays previously established for evaluation of the efficacy of antivirals against hepatitis C virus^31,32^. VeroE6 cells were plated at 10,000 cells per well of poly-d-lysine-coated 96-well plates (Thermo Fisher Scientific, Rochester, NY, USA). For pretreatment assays, the next day, medium was exchanged to medium containing extracts or compounds adding 50 µL per well. After 1.5 h of incubation at 37 °C and 5% CO_2_, cells were inoculated with SARS-CoV-2/human/Denmark/DK-AHH1/2020 at MOI 0.0016 by adding 50 µL of diluted virus stock per well, resulting in the specified concentrations of extracts or compounds. For treatment assays, the next day, medium was exchanged by adding 50 µL of fresh medium per well. Then, cells were inoculated with SARS-CoV-2/human/Denmark/DK-AHH1/2020 at MOI 0.0016 by adding 50 µL of diluted virus stock per well. MOI were chosen to yield obviously infected cultures with counts of infected cells in the target range given in the section on immunostaining and evaluation of 96-well plates below. In addition, MOI were selected to avoid virus induced cytopathic effects during the assay. One hour after viral inoculation and incubation at 37 °C with 5% CO_2_, 50 µL of medium containing extracts or compounds were added resulting in the specified concentrations; alternatively, 50 µL of medium containing diluent (DMSO) were added resulting in the specified dilutions. For both assays, in each independent experiment, each concentration/dilution was tested in seven replicates; 14 infected and nontreated as well as 12 noninfected and nontreated control wells were included in each assay. After 48 ± 2 h incubation at 37 °C and 5% CO_2_, cultures were immunostained for SARS-CoV-2 spike glycoprotein and evaluated as described below.

### Concentration–response antiviral treatment assay in Huh7.5 cells

Huh7.5 cells were plated at 8000 cells per well of flat bottom 96-well plates (Thermo Fisher Scientific, Roskilde, Denmark). The next day, cells were inoculated with SARS-CoV-2/human/Denmark/DK-AHH1/2020 at MOI 0.0198 by adding 50 µL of diluted virus stock per well. MOI were chosen to yield obviously infected cultures with counts of infected cells in the target range given in the section on immunostaining and evaluation of 96-well plates below. In addition, MOI were selected to avoid virus induced cytopathic effects during the assay. Directly after viral inoculation, 50 µL of medium containing extracts or compounds were added resulting in the specified concentrations; alternatively, 50 µL of medium containing diluent (DMSO) were added resulting in the specified dilutions. In each independent experiment, each concentration was tested in seven replicates; 14 infected and nontreated as well as 12 noninfected and nontreated control wells were included in the assay. After 72 ± 2 h incubation at 37 °C and 5% CO_2_, cultures were immunostained for SARS-CoV-2 spike glycoprotein and evaluated as described below.

### Concentration–response antiviral treatment assay in A549-hACE2 cells

A549-hACE2 cells were plated at 10,000 cells per well of flat bottom 96-well plates (Thermo Fisher Scientific, Roskilde, Denmark). The next day, cells were inoculated with SARS-CoV-2/human/Denmark/DK-AHH1/2020 at MOI 0.0025 by adding 50 µL of diluted virus stock per well. MOI were chosen to yield obviously infected cultures with counts of infected cells in the target range given in the section on immunostaining and evaluation of 96-well plates below. In addition, MOI were selected to avoid virus induced cytopathic effects during the assay. Directly after viral inoculation, 50 µL of medium containing extracts or compounds were added resulting in the specified concentrations; alternatively, 50 µL of medium containing diluent (DMSO) were added resulting in the specified dilutions. In each independent experiment, each concentration was tested in seven replicates; 14 infected and nontreated as well as 12 noninfected and nontreated control wells were included in the assay. After 48 ± 2 h incubation at 37 °C and 5% CO_2_, cultures were immunostained for SARS-CoV-2 spike glycoprotein and evaluated as described below.

### Time-of-addition experiment with artesunate

A549-hACE2 cells were plated at 10,000 cells per well of flat bottom 96-well plates (Thermo Fisher Scientific, Roskilde, Denmark). The next day, cells were inoculated with SARS-CoV-2/human/Denmark/DK-AHH1/2020 at MOI 0.005 by adding 50 µL of diluted virus stock per well. The virus containing supernatants were removed after 2 h incubation, and wells were washed once with 130 µL PBS. Directly after, 50 µL of medium were added. At different timepoints during the experiment, 50 µL of medium containing artesunate were added resulting in a final concentration of 14 µg/mL: 0 h post inoculation, addition at the time of viral inoculation with presence of the drug during the 2 h viral infection phase; 2 h, addition 2 h post inoculation, immediately after the 2 h viral infection phase; 4 h and 6 h, addition 4 h and 6 h post inoculation, respectively. During the experiment, cultures were incubated at 37 °C and 5% CO_2_. 48 ± 2 h after inoculation, cultures were immunostained for SARS-CoV-2 spike glycoprotein and evaluated as described below. 96-well images from this experiment in A549-hACE2 cells are shown in Figure S11.

### Immunostaining and evaluation of 96-well plates for concentration–response antiviral treatment and time-of-addition assays

Cells were fixed and virus was inactivated by immersion of plates in methanol (J.T.Baker, Gliwice, Poland) for 20 min. Unless specified otherwise, immunostaining was done at room temperature. Plates were washed twice with PBS (Sigma, Gillingham, UK) containing 0.1% Tween-20 (Sigma, Saint Louis, Missouri, USA). Endogenous peroxidase activity was blocked by incubation with 3% H_2_O_2_ for ten minutes followed by two washes with PBS containing 0.1% Tween-20 and blocking with PBS containing 1% bovine serum albumin (Roche, Mannheim, Germany) and 0.2% skim milk powder (Easis, Aarhus, Denmark) for 30 min. Following removal of blocking solution, plates were incubated with primary antibody SARS-CoV-2 spike chimeric monoclonal antibody (Sino Biological #40150-D004, Beijing, China) diluted 1:5000 in PBS containing 1% bovine serum albumin and 0.2% skim milk powder overnight at 4 ℃. Following two washes with PBS containing 0.1% Tween-20, plates were incubated with secondary antibody F(ab')2-Goat anti-Human IgG Fc Cross-Adsorbed Secondary Antibody, HRP (Invitrogen #A24476, Carlsbad, CA, USA) or Goat F(ab')2 Anti-Human IgG—Fc (HRP), pre-adsorbed (Abcamab#98595, Cambridge, UK) diluted 1:2000 in PBS containing 1% bovine serum albumin and 0.2% skim milk powder for 2 h. Following two washes with PBS containing 0.1% Tween-20, SARS-CoV-2 spike glycoprotein was visualized using DAB substrate (Immunologic # BS04-110, Duiven, Netherlands). Spike protein positive cells were counted automatically using an ImmunoSpot series 5 UV analyzer (CTL Europe GmbH, Bonn, Germany) as described^31-33^. The average count of 12 noninfected nontreated control wells, which was usually < 50, was subtracted from the count of each infected well. Counts recorded in each infected treated well were related to the average count of 14 infected nontreated control wells to calculate % residual infectivity. Datapoints included in figures are means of seven replicates from one independent experiment with standard errors of the means (SEM). Sigmoidal dose response curves were fitted and EC_50_ values were calculated with GraphPad Prism 8.0.0 using a bottom constraint of 0 and the formula Y = Top/(1 + 10^((LogEC_50_-X)*HillSlope)). The MOI for infection was chosen aiming at on average 3000–4000 counts per well for VeroE6 cells and A549-hACE2 cells, and on average 300–600 counts per well for the less permissive Huh7.5 cells in infected nontreated control wells upon termination of the respective assays. Representative 96-well images from assays in VeroE6 cells are shown in Figure S3 and representative images of single wells are show in Figure S4.

### Cell viability assays in VeroE6, Huh7.5 and A549-hACE2 cells

To evaluate cytotoxic effects of the tested extracts, compounds, and diluents (DMSO), cell viability was monitored using the CellTiter 96 AQueous One Solution Cell Proliferation Assay (Promega, Madison, WI, USA). VeroE6 cells, Huh7.5 cells or A549-hACE2 cells were plated at 10,000, 8000 or 10,000 cells per well of flat bottom 96-well plates, respectively (Thermo Fisher Scientific, Roskilde, Denmark). The next day, medium was exchanged to contain specified concentrations of extracts or compounds or dilutions of DMSO adding 100 µL per well. Each concentration or dilution was tested in 3 replicates; at least 6 nontreated control wells were included in the assay. For VeroE6 cells and A549-hACE2 cells, after 48 ± 2 h, and for Huh7.5 cells, after 72 ± 2 h of incubation at 37 °C and 5% CO_2_, 20 µL CellTiter 96 AQueous One Solution Reagent was added per well and plates were incubated for 1.5 to 2 h at 37 °C and 5% CO_2_, prior to recording absorbance at 492 nm using a FLUOstar OPTIMA 96-well plate reader (BMG LABTECH, Offenburg, Germany). Absorbance recorded in each well was related to the average absorbance of nontreated control wells to calculate the percentage of cell viability. Datapoints included in figures are means of triplicates from one independent experiment with SEM. Sigmoidal dose response curves were fitted and median cytotoxic concentration (CC_50_) values were calculated with GraphPad Prism 8.0.0 using a bottom constraint of 0 and the formula Y = Top/(1 + 10^((LogCC_50_-X) * HillSlope)) as further specified in Figures S5, S7, and S9. To rule out cytotoxic effects at the concentrations selected based on cell viability assays in the presence of viral infection, culture wells in antiviral assays were manually inspected in the light microscope.

## Supplementary Information

Supplementary Information.

## Abbreviations

API: Active pharmaceutical ingredients
CC_50_: Median cytotoxic concentration
COVID-19: Coronavirus disease 2019
DMSO: Dimethylsulfoxide
EC_50_: Median effective concentration
FBS: Fetal bovine serum
FDA: Food and Drug Administration
hACE2: human angiotensin-converting enzyme 2
IF: Immunofluorescence
MOI: Multiplicity of infection
PBS: Phosphate buffered saline
SARS-CoV-2: Severe acute respiratory syndrome coronavirus 2
SD: Standard deviation
SEM: Standard error of the mean
SI: Selectivity index
