# Supplementary material for: In vitro efficacy of artemisinin-based treatments against SARS-CoV-2
Source: Sci Rep. 2021 Jul 16;11:14571. doi: 10.1038/s41598-021-93361-y (PMC8285423; doi:10.1038/s41598-021-93361-y)
Supplement: Supplementary file 1 — Supplementary Information. [file 41598_2021_93361_MOESM1_ESM.docx]

# *In vitro* efficacy of Artemisinin-based treatments against SARS-CoV-2

Yuyong Zhou,^$1, 2^ Kerry Gilmore,^$3^ Santseharay Ramirez,^1, 2^ Eva Settels, ^3^ Karen A. Gammeltoft,^1, 2^ Long V. Pham,^1, 2^ Ulrik Fahnøe,^1, 2^ Shan Feng,^1, 2^ Anna Offersgaard,^1, 2^ Jakob Trimpert,^4^ Jens Bukh,^1, 2^ Klaus Osterrieder, ^*4, 5^ Judith M. Gottwein,*^£1, 2^ Peter H. Seeberger*^£3, 6^

^1^ Copenhagen Hepatitis C Program (CO-HEP), Department of Infectious Diseases, Copenhagen University Hospital-Hvidovre, Kettegård Alle 30, 2650, Hvidovre, Denmark

^2^ CO-HEP Department of Immunology and Microbiology, Faculty of Health and Medical Sciences, University of Copenhagen, Blegdamsvej 3B, 2200, Copenhagen, Denmark

^3^ Max Planck Institute for Colloids and Interfaces, Am Mühlenberg 1, 14476 Potsdam, Germany

^4^ Institute for Virology, Freie Universität Berlin, Robert von Ostertag-Str. 7-13, 14163, Berlin, Germany

^5^ Department of Infectious Diseases and Public Health, Jockey Club College of Veterinary Medicine and Life Sciences, City University of Hong Kong, Kowloon Tong, Hong Kong

^6^ Institute of Chemistry and Biochemistry, Freie Universität Berlin, Arnimallee 22, 14195, Berlin, Germany

^$^ These authors contributed equally: Yuyong Zhou and Kerry Gilmore.

* Corresponding Authors: no34@cornell.edu, jgottwein@sund.ku.dk, and Peter.seeberger@mpikg.mpg.de

^£^ These authors jointly supervised this work: Judith M. Gottwein and Peter H. Seeberger.

**SUPPLEMENTARY INFORMATION**

Contents

[*In vitro* efficacy of Artemisinin-based treatments against SARS-CoV-2 1](#_Toc72311800)

[1. Reagents and Origins of Natural Substances 2](#_Toc72311801)

[2. Extraction of *A. annua* leaves 3](#_Toc72311802)

[General procedure A: Extraction using absolute ethanol 3](#_Toc72311803)

[General procedure B: Extraction using distilled water 3](#_Toc72311804)

[3. Extracts and Compounds for Treatment Assays 4](#_Toc72311805)

[4. Efficacy of *A. annua* extracts and artemisinin against SARS-CoV-2 in a plaque-reduction assay in VeroE6 cells 8](#_Toc72311806)

[5. Effect of artemisinin-based treatment and diluents on SARS-CoV-2 infection and cell viability *in vitro* using concentration response antiviral treatment assays 11](#_Toc72311807)

[5.1 Concentration response antiviral treatment assays in VeroE6 cells 11](#_Toc72311808)

[5.2 Influence of artemisinin-based treatment on VeroE6 cell viability 14](#_Toc72311809)

[5.3 Influence of diluent DMSO on SARS-CoV-2 infection and cell viability in VeroE6 cells 16](#_Toc72311812)

[5.4 Influence of artemisinin-based treatment on Huh7.5 cell viability 17](#_Toc72311813)

[5.5 Influence of diluent DMSO on SARS-CoV-2 infection and cell viability in Huh7.5 cells 18](#_Toc72311814)

[5.6 Influence of artemisinin-based treatment on A549-hACE2 cell viability 19](#_Toc72311815)

[6. Time-of-addition experiment with artesunate in A549-hACE2 cells 21](#_Toc72311816)

[7. References 23](#_Toc72311817)

# 1. Reagents and Origins of Natural Substances

Solvents were obtained from commercial suppliers and used without further purification. Dried *Artemisia annua* leaves were obtained from ArtemiLife Inc. All crops were grown and harvested in 2019 and air-dried in partial sunlight. *Artemisia annua* has been grown under standardized conditions developed by researchers at ArtemiLife. Transplants are grown from a cultivated seedline in greenhouses for approximately seven weeks. Transplants are then distributed to growers in the surrounding region of Lexington, Kentucky, USA. Planting is achieved using widely available transplanter equipment in prepared fields. Crops are fertilized, watered, and manually weeded. Dihydroartemisinic acid and artemisinin content is monitored throughout the season via HPLC analysis of leaf extracts. Harvest is determined by content concentrations. Plants are cut and dried in tobacco barns. Leaves are manually separated, and samples are packed and stored under ambient conditions. For further details, please contact ArtemiLife.

Stripped leaves were ground to pieces approximately ≤ 1 mm and stored in nylon bags at room temperature and used without further manipulation. Artemisinin was either previously prepared and purified by crystallization using published protocols or purchased (Sigma, Saint Louis, Missouri, USA). Artesunate was purchased (Selleckchem, Houston, Texas, USA or TCI, Eschborn, Germany). Artemether was purchased (Selleckchem, Houston, USA). Crystals were ground prior to use. Compounds were dissolved in DMSO and frozen.^S1^

# 2. Extraction of *A. annua* leaves

## General procedure A: Extraction using absolute ethanol

A magnetic stir bar (6 cm) and absolute ethanol (250 mL, VWR) were added to an Erlenmeyer flask (500 mL). The stir bar was rotated at 550 rpm using a Heidolph MR HEI-END heated stir plate. The solvent was heated to 50 ˚C using the hot plate. Dried leaf material (50 g, Figure S1) was added to the ethanol using a powder funnel and allowed to stir for 200 minutes. The samples were then removed from the stir plate and vacuum filtered using a fritted filter with filter paper. The solid material was washed with room temperature ethanol until the liquid exiting the filter was clear. The solution was then dried using a rotary evaporator and further dried for at least two hours on high vacuum.

## General procedure B: extraction using distilled water

A magnetic stir bar (6 cm) and distilled water (250 mL, VWR) were added to an Erlenmeyer flask (500 mL). The stir bar was rotated at 550 rpm using a Heidolph MR HEI-END heated stir plate. The solvent was heated to 50 ˚C using the hot plate. Dried leaf material (25 g) was added to the temperature-stable water using a powder funnel and allowed to stir for 200 minutes. Note: half the mass of plant material was used as compared to General Procedure A due to the increased absorbance of the water into the plant material. Using 125 mL (desired ratio) or 200 mL of water resulted in a thick sludge as opposed to a freely flowing heterogeneous solution.

The samples were then removed from the stir plate and vacuum filtered using a fritted filter with filter paper. The solid material was washed with room temperature water until the liquid exiting the filter was clear. The solution was then dried using a rotary evaporator and further dried for at least 2 hours on high vacuum.


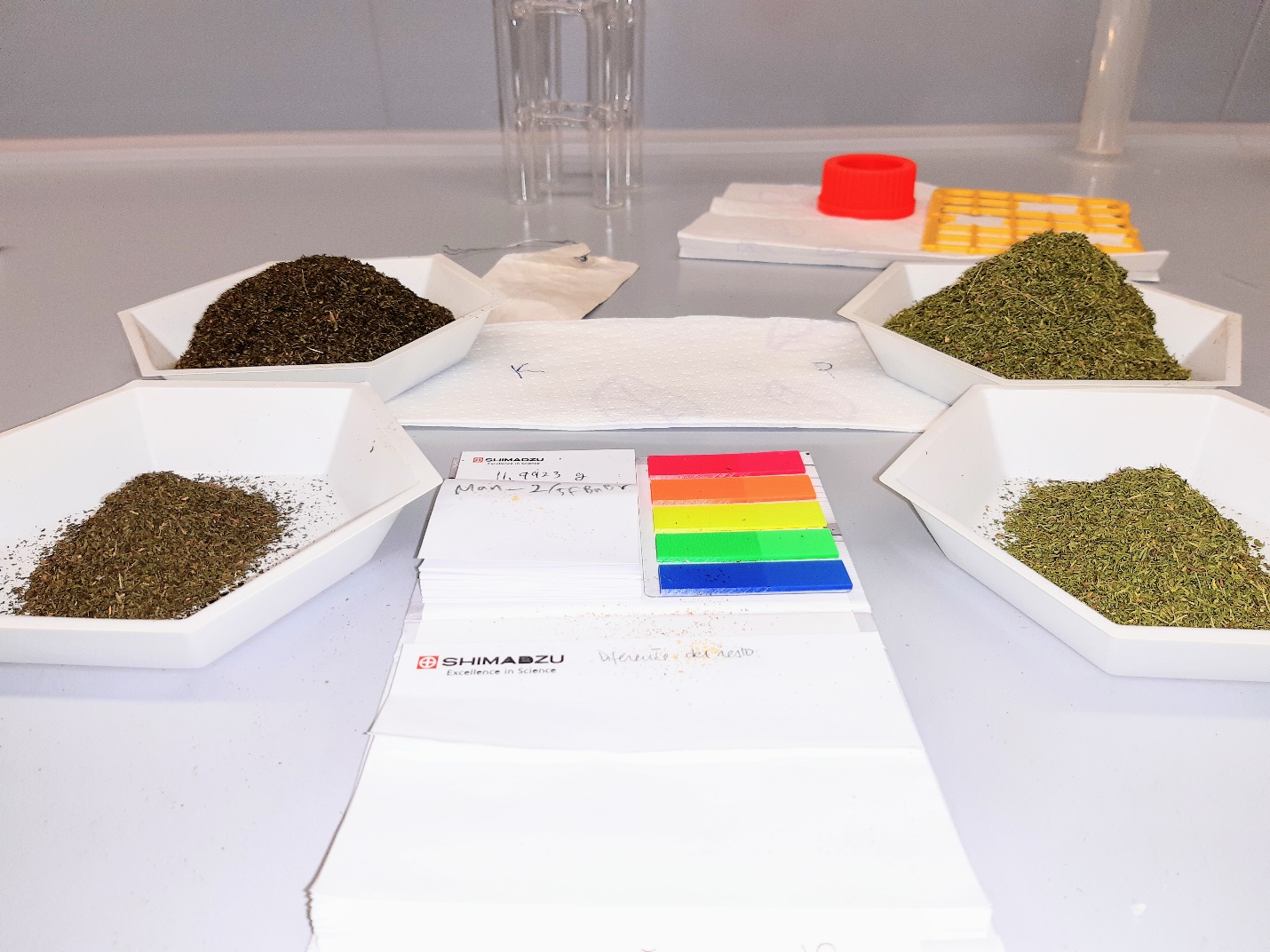


b.

a.

Figure S1: Dried leaves of *Artemisia annua*. a) Plant material (50 g). b) Plant material (4 g). Seedlings were planted in May and plants harvested in September 2019.

**Extraction of *A. annua***

Extracts were prepared following General Procedures A and B.

***A. annua* ethanolic extract**: *A. annua* (50 g) from Lancaster, KY was added to 250 mL of 100% ethanol at 50 ˚C, stirred by magnetic stir bar. After 200 min, the plant material was removed by vacuum filtration (using a glass fritted filter and filter paper) and washed with 100% ethanol. Ethanol was removed from the solution using a rotary evaporator with water bath at 40 ˚C. The flask was further dried for three hours under high vacuum. The dried material (7.404 g) was a dark green, sticky material. The flask was covered with aluminum foil and stored at -30 ˚C prior to sample preparation.

***A. annua* aqueous extract**: *A. annua* (25 g) from Lancaster, KY was added to 250 mL of distilled water at 50 ˚C, stirred by magnetic stir bar. After 200 min, the plant material was removed by vacuum filtration (using a glass fritted filter and filter paper) and washed with distilled water. Water was removed from the solution using a rotary evaporator with water bath at 40 ˚C. The flask was further dried for three hours under high vacuum. The dried material (6.915 g) was a dry, green-brown material. The flask was covered with aluminum foil and stored at -30 ˚C prior to sample preparation.

# Extracts and Compounds for Treatment Assays

Nine samples of extracts and compounds were used in antiviral assays. An overview of the preperations and detailed descriptions of preparations used in specific applications are provided below.

**Overview of preparations**

**Sample 1 – *A. annua* ethanolic extract.** A.annua ethanolic extract (302.1 mg) was dissolved in DMSO (3 mL). Light heating was required for maximal solvation – trace amounts of solid material could be observed. The sample was filtered through a syringe filter (Chromafil xtra RC0.45). One mL was removed with a 1000 µL pipette and delivered to a 1.5 mL eppendorf snap-close vial. Concentration of example sample: ~100 mg/mL. The sample was stored at -20 °C until use.

**Sample 2 – *A. annua* aqueous extract.** A. annua aqueous extract (560.9 mg) was dissolved in DMSO:H_2_O (3:1,8 mL). Light heating was required for maximal solvation – trace amounts of solid material could be observed. The sample was filtered through a syringe filter (Chromafil xtra RC0.45). One mL was removed with a 1000 µL pipette and delivered to a 1.5 mL eppendorf snap-close vial. Concentration of example sample: ~70 mg/mL. The sample was stored at -20 °C until use.

**Sample 3 – Artemisinin.** Artemisinin (250.3 mg) synthesized at MPICI was dissolved in DMSO (1.0 mL). The solution was transferred with a 1000 µL pipette to a 1.5 mL Eppendorf snap-close vial. Concentration of example sample: ~250 mg/mL. The sample was stored at -20 °C until use.

**Sample 4 – Artesunate.** Artesunate (249.2 mg) obtained from TCI (Eschborn, Germany) was dissolved in DMSO (1 mL). Full solvation was obtained with light heating. The solution was transferred to a 1.5 mL Eppendorf snap-close vial. Concentration of example sample: ~250 mg/mL. The sample was stored at -20 °C until use.

**Sample 5 – Artemether.** Artemether (10 mg) obtained from Selleckchem (Houston, Texas, USA) was dissolved in DMSO (180 µL). The solution was transferred to 1.5 mL Eppendorf snap-close vials. Concentration of example sample: ~56 mg/mL. The sample was stored at -80 °C until use.

**Sample 6 – Artemisinin.** Artemisinin (100 mg) obtained from Sigma (Saint Louis, Missouri, USA) was dissolved in DMSO (1900 µL). The solution was transferred to 1.5 mL Eppendorf snap-close vials. Concentration of example sample: ~53 mg/mL. The sample was stored at -80 °C until use.

**Sample 7 – Artesunate.** Artesunate (10 mg) obtained from Selleckchem (Houston, Texas, USA) was dissolved in DMSO (140 µL). The solution was transferred to 1.5 mL Eppendorf snap-close vials. Concentration of example sample: ~71 mg/mL. The sample was stored at -80 °C until use.

**Preparations used in VeroE6 plaque-assays:** (Supplementary Information section 4)

***A. annua* ethanolic extract.** – **Sample 1** as described above.

***A. annua* aqueous extract.** – **Sample 2** as described above.

**Artemisinin. – Sample 3** as described above.

**Preprations used in VeroE6 pre-treatment and treatment assays:** (Figures 2 and 3)

***A. annua* ethanolic extract.** – **Sample 1** as described above.

***A. annua* aqueous extract.** – **Sample 8** was prepared as sample 2 with minor modifications: *A. annua* aqueous extract (470.8 mg) was dissolved in DMSO:H_2_O (3:1,8 mL). Light heating was required for maximal solvation – trace amounts of solid material could be observed. The sample was filtered through a syringe filter (Chromafil xtra RC0.45). One mL was removed with a 1000 µL pipette and delivered to a 1.5 mL eppendorf snap-close vial. Concentration of sample: ~59 mg/mL. The sample was stored at -20 °C until use.

**Artemisinin.** – **Sample 3** as described above.

**Artesunate.** ­­– **Sample 4** as described above.

**Artemether.** – **Sample 5** as described above.

**Preprations used in Huh7.5 treatment assays**: (Figure 4)

**Artemisinin.** – **Sample 3 –**as described above.

**Artesunate.** ­­– **Sample 4** as described above.

**Artemether.** – **Sample 5** as described above.

***A. annua* ethanolic extract.** **– Sample 9** was prepared as sample 1 with minor modifications: *A.annua* ethanolic extract (181.0 mg) was dissolved in DMSO (3 mL). Slight heating was required for maximal solvation – trace amounts of solid material could be observed. The sample was filtered through a syringe filter (Chromafil xtra RC0.45). One mL was removed with a 1000 µL pipette and delivered to a 1.5 mL eppendorf snap-close vial. Concentration of sample: ~60 mg/mL.The sample was stored at -20 °C until use.

**Preprations used in A549-hACE2 treatment assays:** (Figures 5 and 6)

**Artemisinin. – Sample 6** as described above.

**Artesunate.** ­­– **Sample 7** as described above.

**Artemether.** – **Sample 5** as described above.

***A. annua* ethanolic extract.** **– Sample 9** as described above**.**

**Qualitative HPLC analysis of artemisinin and dihydroartemisinic acid in *A. annua* extract**

*A. annua* ethanolic extract was analyzed using an Agilent 1260 Series HPLC system composed of an auto sampler, a binary pump and a column oven. The system was coupled to an evaporative light scattering detector (Agilent Infinity II ELSD) and mass spectrometry detector (Agilent). The extract was dissolved in acetonitrile and filtered using a 0.45 µm cellulose syringe filter prior to analysis. Acetonitrile/water+0.1 vol% formic acid (80/20 v/v) served as mobile phase at a flow rate of 1 mL/min using a Phenomenex Synergi C18 (250 × 4.6 mm, 4.6 µm) column as stationary phase at 25°C. A sample of 30 µL was injected and analyzed. Two detectors were used: Detector I: Evaporative light scattering detector Agilent 1260 Infinity II ELSD at an evaporator temperature of 40 °C and a nebulizer temperature of 40 °C with a gas flow rate 1.60 L/min and Detector II: Mass spectrometry detector Agilent LC/MSD XT with Jetstream source.

The HPLC chromatogram shows artemisinin (at 4.922 min) and dihydroartemisinic acid (at 7.627 min) as determined by mass spectrometry.


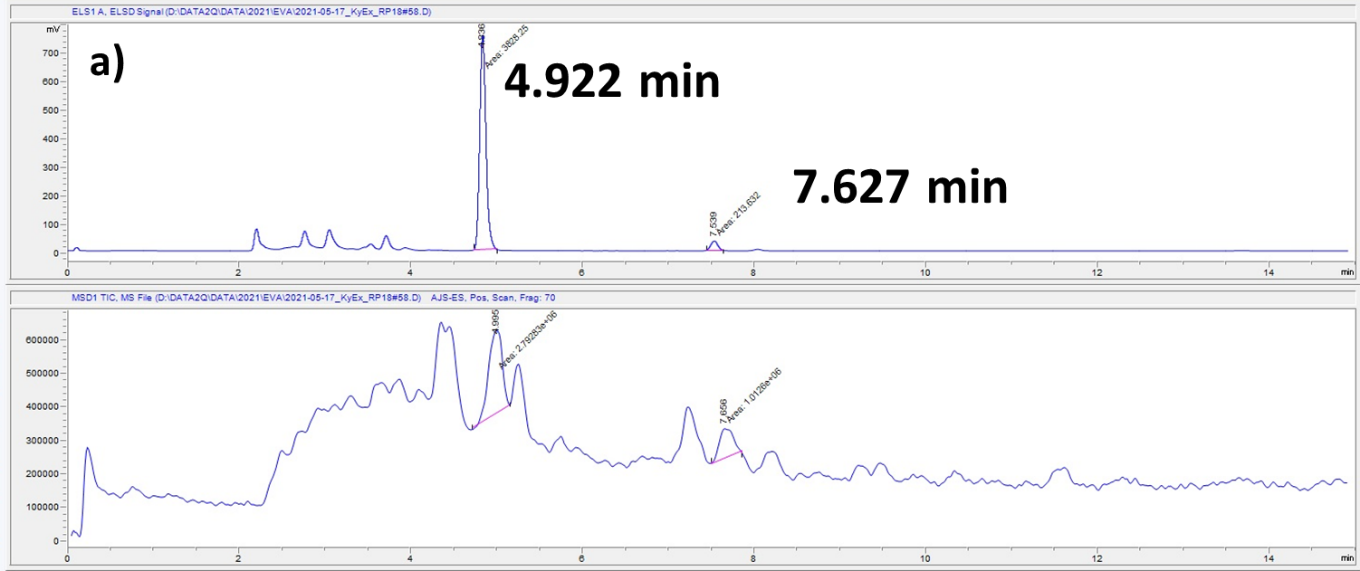


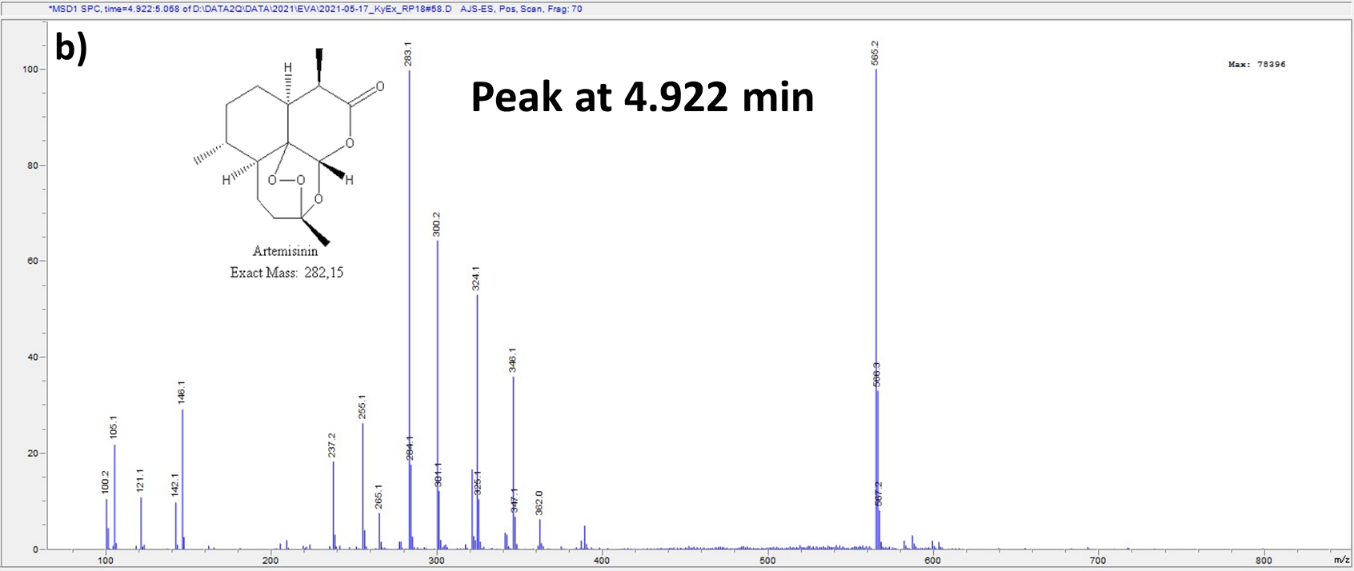


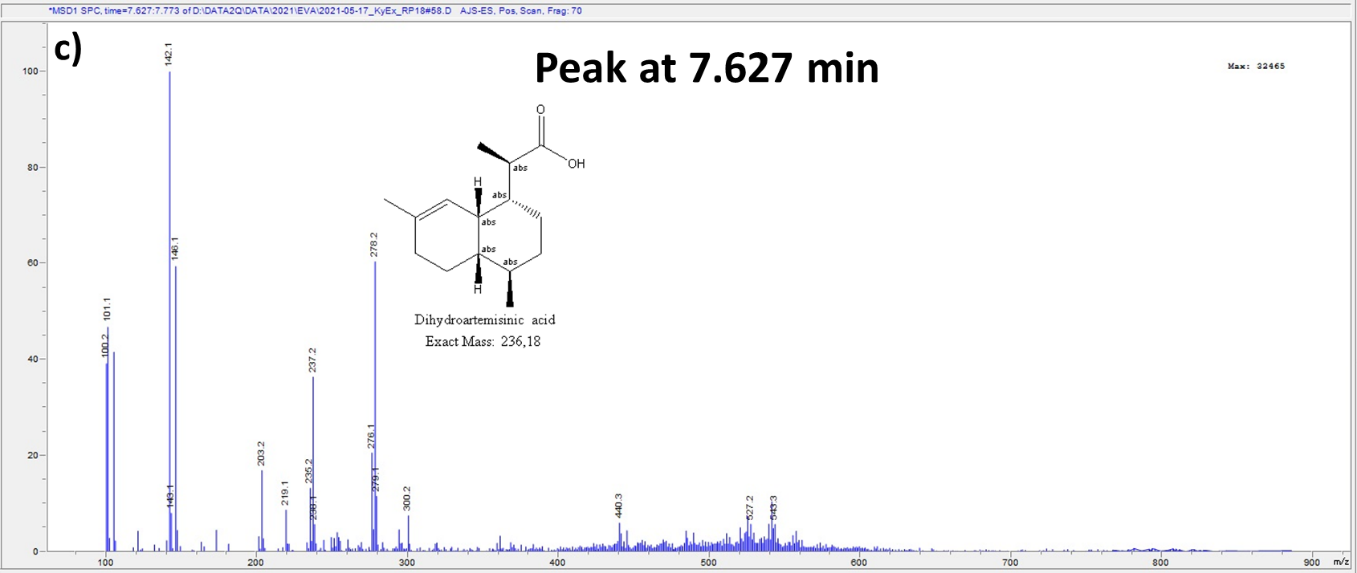


**Figure S2:** Plant material was prepared and extracted with ethanol following General Procedure A to yield “Sample 1”. The HPLC analysis was performed as described above.

# 4. Efficacy of *A. annua* extracts and artemisinin against SARS-CoV-2 in a plaque-reduction assay in VeroE6 cells

Efficacy of *A. annua* extracts and artemisinin was evaluated using plaque-reduction assays in VeroE6 cells in two screening rounds. The data obtained in these assays are shown in Table S1 to S8 below.

Screening round 1:

Table S1: *A. annua* ethanolic extract data for pretreatment screening using Vero E6 cells. The time is the pretreatment time of the sample prior to virus infection. Values reported are the number of plaques observed divided by the average number of plaques in the negative control multiplied by 100. An overview on the number of plaques in the negative control is found it Table S7.

|  | **Sample concentrations (mg/mL)** | | |
| --- | --- | --- | --- |
|  | 200 | 20 | 2 |
|  | **Log concentrations** | | |
| time (min) | 2.3 | 1.3 | 0.3 |
| 15 | 43 | 76 | 83 |
| 30 | 61 | 76 | 85 |
| 60 | 42 | 71 | 60 |

Table S2: *A. annua* aqueous extract data for pretreatment screening using Vero E6 cells. The time is the pretreatment time of the sample prior to virus infection. Values reported are the number of plaques observed divided by the average number of plaques in the negative control multiplied by 100. An overview on the number of plaques in the negative control is found it Table S7.

|  | **Sample concentrations (mg/mL)** | | |
| --- | --- | --- | --- |
|  | 260 | 26 | 2.6 |
|  | **Log concentrations** | | |
| time (min) | 2.4 | 1.4 | 0.4 |
| 15 | 69 | 89 | 852 |
| 30 | 57 | 101 | 110 |
| 60 | 35 | 39 | 66 |

Table S3: Artemisinin data for pretreatment screening using Vero E6 cells. The time is the pretreatment time of the sample prior to virus infection. Values reported are the number of plaques observed divided by the average number of plaques in the negative control multiplied by 100. An overview on the number of plaques in the negative control is found it Table S7.

N.A. = not available.

|  | **Sample concentrations (mg/mL)** | | |
| --- | --- | --- | --- |
|  | 220 | 22 | 2.2 |
|  | **Log concentrations** | | |
| time (min) | 2.3 | 1.3 | 0.3 |
| 15 | N.A. | 76 | 67 |
| 30 | N.A. | 86 | 95 |
| 60 | N.A. | 42 | 57 |

Screening round 2:

Table S4: *A. annua* ethanolic extract data for pretreatment screening using Vero E6 cells. The time is the pretreatment time of the sample prior to virus infection. Values reported are the number of plaques observed divided by the average number of plaques in the negative control multiplied by 100. An overview on the number of plaques in the negative control is found it Table S8.

|  | **Sample concentrations (mg/mL)** | | | |
| --- | --- | --- | --- | --- |
|  | 200 | 20 | 2 | 0.2 |
|  | **Log concentrations** | | | |
| time (min) | 2.3 | 1.3 | 0.3 | -0.7 |
| 15 | 39 | 54 | 72 | 82 |
| 30 | 46 | 65 | 69 | 97 |
| 60 | 66 | 80 | 85 | 94 |
| 120 | 71 | 86 | 86 | 105 |

Table S5: *A. annua* aqueous extract data for pretreatment screening using Vero E6 cells. The time is the pretreatment time of the sample prior to virus infection. Values reported are the number of plaques observed divided by the average number of plaques in the negative control multiplied by 100. An overview on the number of plaques in the negative control is found it Table S8.

|  | **Sample concentrations (mg/mL)** | | | |
| --- | --- | --- | --- | --- |
|  | 260 | 26 | 2.6 | 0.26 |
|  | **Log concentrations** | | | |
| time (min) | 2.415 | 1.415 | 0.415 | -0.585 |
| 15 | 49 | 71 | 96 | 100 |
| 30 | 52 | 62 | 69 | 110 |
| 60 | 20 | 59 | 73 | 103 |
| 120 | 29 | 46 | 83 | 86 |

Table S6: Artemisinin data for pretreatment screening using Vero E6 cells. The time is the pretreatment time of the sample prior to virus infection. Values reported are the number of plaques observed divided by the average number of plaques in the negative control multiplied by 100. An overview on the number of plaques in the negative control is found it Table S8.

|  | **Sample concentrations (mg/mL)** | | | |
| --- | --- | --- | --- | --- |
|  | 220 | 22 | 2.2 | 0.22 |
|  | **Log concentrations** | | | |
| time (min) | 2.3 | 1.3 | 0.3 | -0.7 |
| 15 | 60 | 80 | 84 | 96 |
| 30 | 68 | 78 | 79 | 105 |
| 60 | 74 | 94 | 77 | 96 |
| 120 | 82 | 103 | 96 | 103 |

Table S7: Number of plaques in the negative (DMSO) control in screening round 1.

| Entry | # of plaques |
| --- | --- |
| 1 | 248 |
| 2 | 246 |
| 3 | 270 |
| 4 | 212 |
| 5 | 228 |
| Average | 240.8 |

Table S8: Number of plaques in the negative (DMSO) control in screening round 2.

| Entry | # of plaques |
| --- | --- |
| 1 | 110 |
| 2 | 118 |
| 3 | 130 |
| 4 | 132 |
| 5 | 140 |
| 6 | 148 |
| Average | 129.67 |

# 5. Effect of artemisinin-based treatment and diluents on SARS-CoV-2 infection and cell viability *in vitro* using concentration response antiviral treatment assays

# 5.1 Concentration response antiviral treatment assays in VeroE6 cells

**
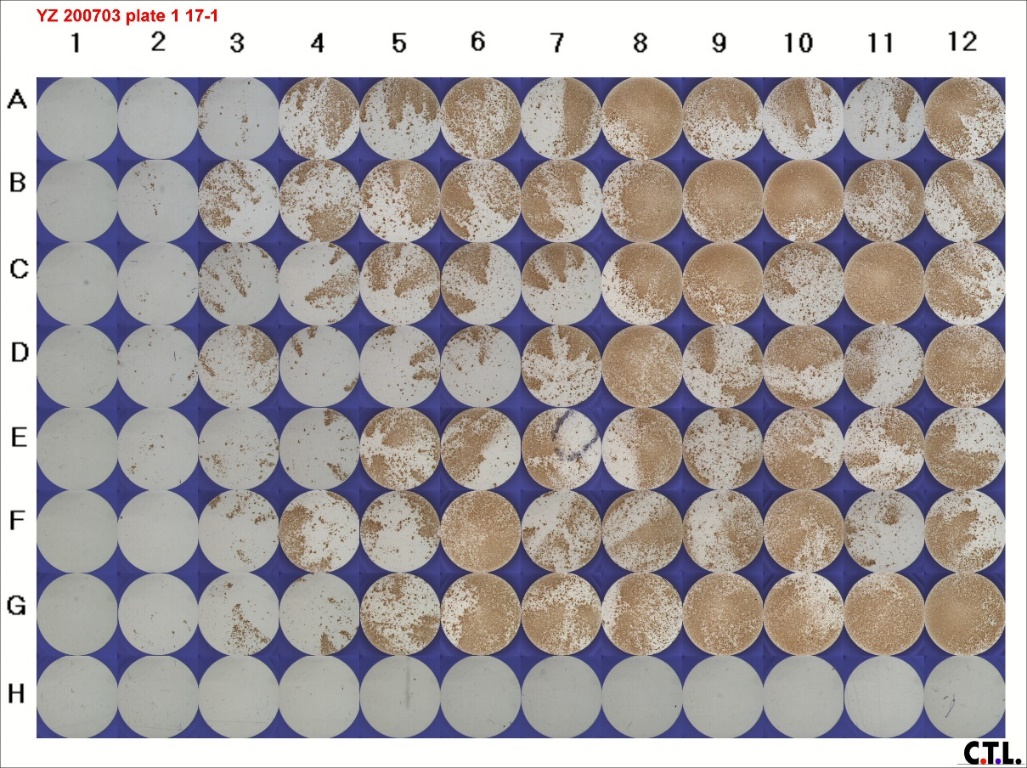
**

**
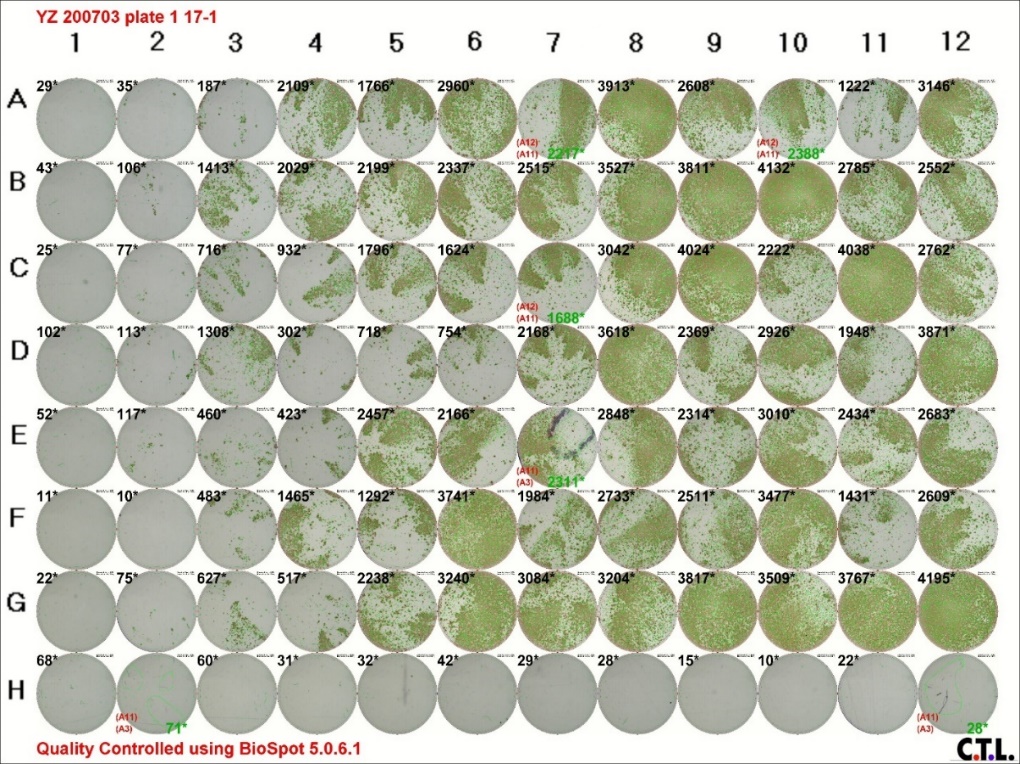
**

**Figure S3. Representative 96-well images from concentration response antiviral treatment assays in VeroE6 cells.** A concentration response antiviral treatment assay was carried out in VeroE6 cells as described in Materials and Methods using the *A. annua* ethanolic extract. An image of the complete 96-well plate is shown prior to (top image) and following (bottom image) automated counting of single SARS-CoV-2 spike glycoprotein positive cells. Row H: non-infected controls (12 replicates). Column 11-12 rows A-G infected non-treated controls (14 replicates). Column 1 to 10 contained decreasing concentrations of extract with 7 replicates per concentration.

**
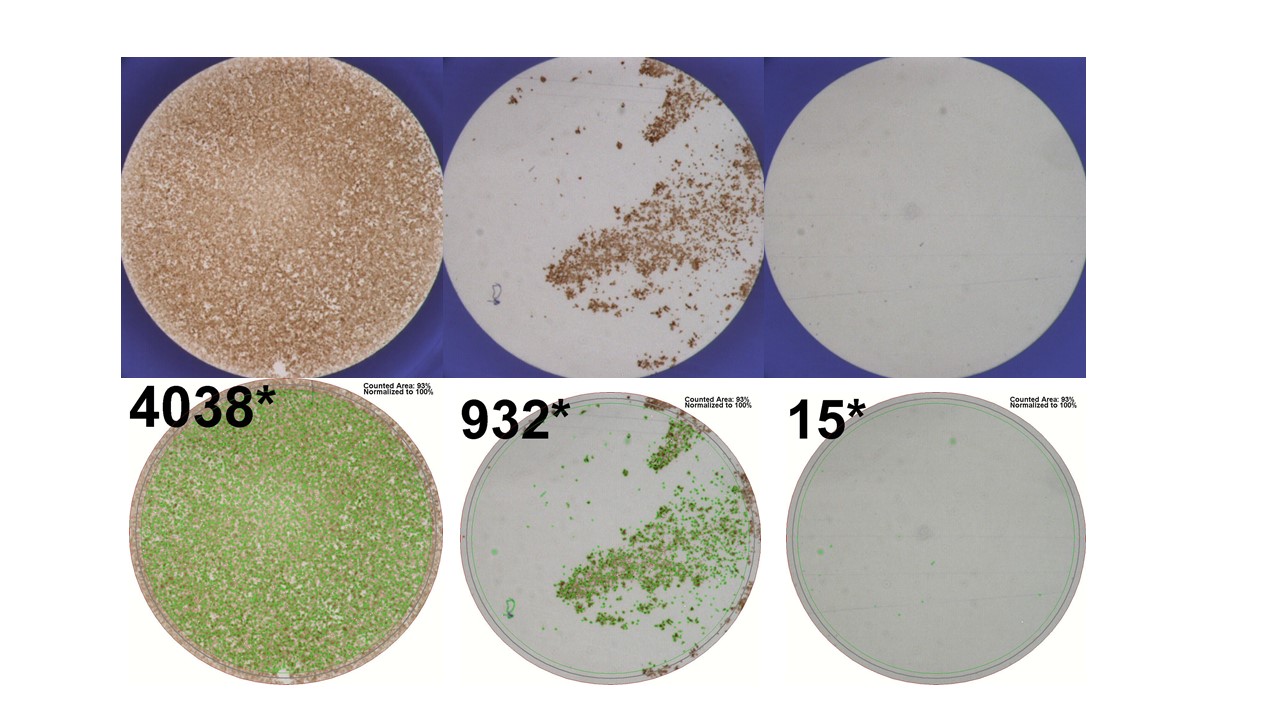
**

**Figure S4. Representative single-well images from concentration response antiviral treatment** **assays in VeroE6 cells.** A concentration response antiviral treatment assay was carried out in VeroE6 cells as described in Materials and Methods using the *A. annua* ethanolic extract. Representative images of single wells showing different degrees of SARS-CoV-2 infection are shown prior to (first row) and following (second row) counting of single SARS-CoV-2 spike glycoprotein positive cells. Single-well images are derived from Figure S3 wells C11, C4, and H9.

# 5.2 Influence of artemisinin-based treatment on VeroE6 cell viability

#

**Figure S5. Calculation of CC_50_ based on cell viability assays in VeroE6 cells.** Cell viability assays were carried out as described in Materials and Methods using the *A. annua* ethanolic extract (A), *A. annua* aqueous extract (B), or compounds artemisinin (C), artesunate (D), and artemether (E). All datapoints are shown in Figures 2 and 3 of the manuscript. To facilitate fitting of sigmoidal dose response curves and calculation of CC_50_ values, of the datapoints providing the lower plateau of the curve, only the datapoint at the lowest concentration was included in the analysis. Sigmoidal dose response curves were fitted in GraphPad Prism 8.0.0 using a bottom constraint of 0 and the formula Y= Top/(1+10^((LogEC_50_-X)*HillSlope)).

# 5.3 Influence of diluent DMSO on SARS-CoV-2 infection and cell viability in VeroE6 cells

**Figure S6. Effect of DMSO on SARS-CoV-2 and cell viability in VeroE6 cells.** VeroE6 cells seeded the previous day in 96-well plates were infected with SARS-CoV-2 and after 1 hour incubation treated with the specified dilutions of DMSO. After a 2-day incubation, infected cells were visualized by immunostaining for SARS-CoV-2 spike glycoprotein and counted automatically as described in Materials and Methods. % residual infectivity for individual wells was calculated by relating counts of infected treated wells to the mean count of 14 infected nontreated control wells. Datapoints (red dots) are means of seven replicates with standard error of the means (SEM). Sigmoidal dose response curve (red line) was fitted in GraphPad Prism as described in Materials and Methods. % Cell viability was determined in replicate assays without infection with SARS-CoV-2 as described in Materials and Methods. Datapoints (blue triangles) are means of three replicates with SEM.

# 5.4 Influence of artemisinin-based treatment on Huh7.5 cell viability

**Figure S7. Calculation of CC_50_ based on cell viability assays in Huh7.5 cells.** Cell viability assays were carried out as described above using the *A. annua* ethanolic extract (A), or compounds artemisinin (B), artesunate (C), and artemether (D). All datapoints are shown in Figure 4 of the manuscript. To facilitate fitting of sigmoidal dose response curves and calculation of CC_50_ values, of the datapoints providing the lower plateau of the curve, only the datapoint at the lowest concentration was included in the analysis. Sigmoidal dose response curves were fitted in GraphPad Prism 8.0.0 using a bottom constraint of 0 and the formula Y= Top/(1+10^((LogEC_50_-X)*HillSlope)).

# 5.5 Influence of diluent DMSO on SARS-CoV-2 infection and cell viability in Huh7.5 cells

**Figure S8. Effect of DMSO on cell viability and SARS-CoV-2 in Huh7.5 cells.** Huh7.5 cells seeded the previous day in 96-well plates were infected with SARS-CoV-2 and directly treated with the specified dilutions of DMSO. After a 3-day incubation, infected cells were visualized by immunostaining for SARS-CoV-2 spike glycoprotein and counted automatically as described in Materials and Methods. % residual infectivity for individual wells was calculated by relating counts of infected treated wells to the mean count of 14 infected nontreated control wells. Datapoints (red dots) are means of seven replicates with SEM. Sigmoidal dose response curve (red line) was fitted in GraphPad Prism as described in Materials and Methods. % Cell viability was determined in replicate assays without infection with SARS-CoV-2 as described in Materials and Methods. Datapoints (blue triangles) are means of three replicates with SEM.

#

# 5.6 Influence of artemisinin-based treatment on A549-hACE2 cell viability

**Figure S9. Calculation of CC_50_ based on cell viability assays in A549-hACE2 cells.** Cell viability assays were carried out as described above using the *A. annua* ethanolic extract (A), or compounds artemisinin (B), artesunate (C), and artemether (D). Sigmoidal dose response curves were fitted in GraphPad Prism 8.0.0 using a bottom constraint of 0 and the formula Y= Top/(1+10^((LogEC_50_-X)*HillSlope)).

**5.7 Influence of diluent DMSO on SARS-CoV-2 infection and cell viability in** **A549-hACE2 cells**

**Figure S10. Effect of DMSO on cell viability and SARS-CoV-2 in A549-hACE2 cells.** A549-hACE2 cells seeded the previous day in 96-well plates were infected with SARS-CoV-2 and directly treated with the specified dilutions of DMSO. After a 2-day incubation, infected cells were visualized by immunostaining for SARS-CoV-2 spike glycoprotein and counted automatically as described in Materials and Methods. % residual infectivity for individual wells was calculated by relating counts of infected treated wells to the mean count of 14 infected nontreated control wells. Datapoints (red dots) are means of seven replicates with SEM. Sigmoidal dose response curve (red line) was fitted in GraphPad Prism as described in Materials and Methods. % Cell viability was determined in replicate assays without infection with SARS-CoV-2 as described in Materials and Methods. Datapoints (blue triangles) are means of three replicates with SEM.

# 6. Time-of-addition experiment with artesunate in A549-hACE2 cells

**
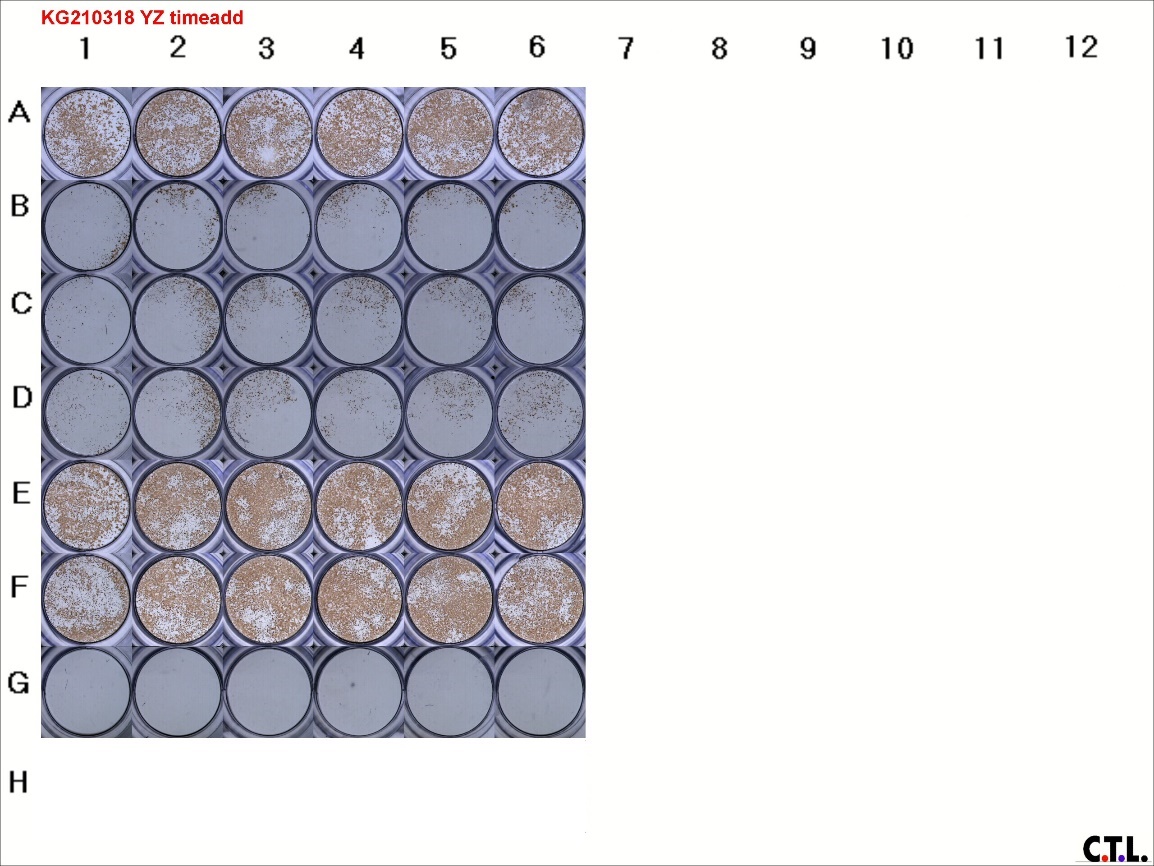

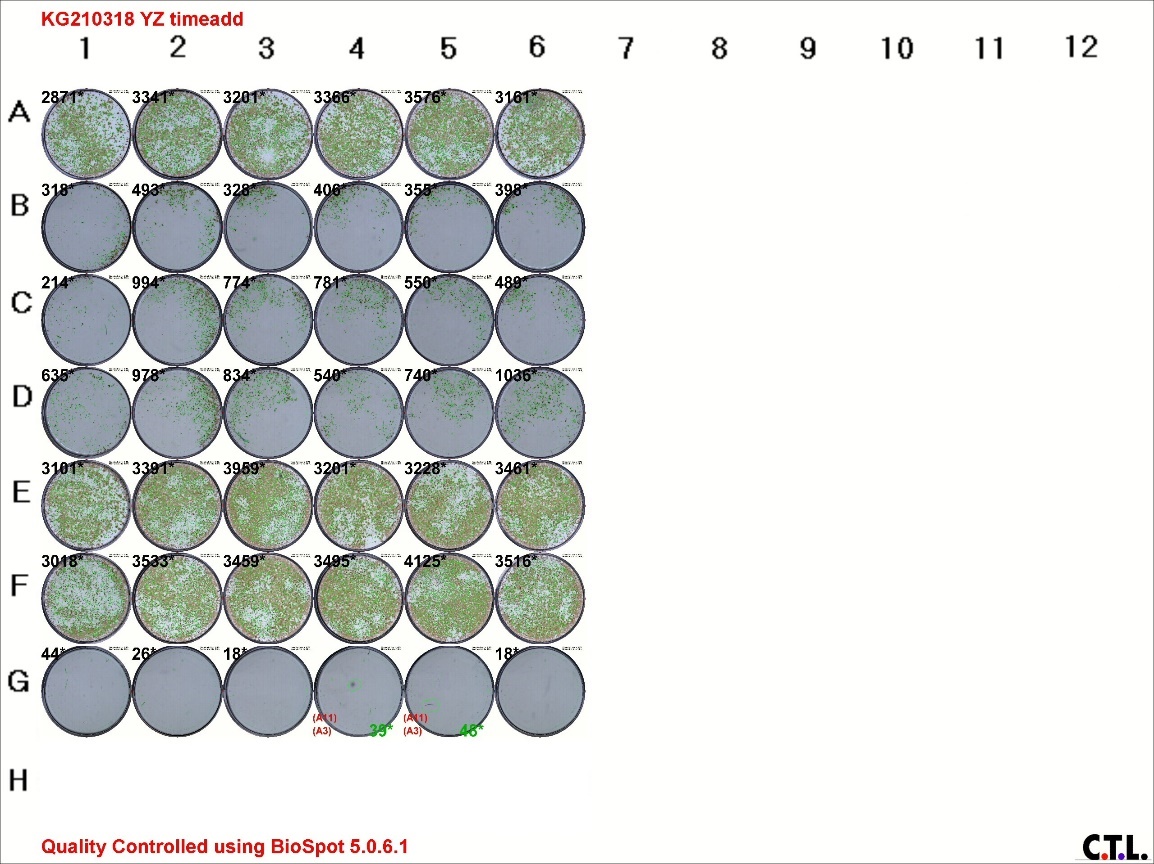
**

**Figure S11. 96-well images from time-of-addition experiment with artesunate in A549-hACE2 cells.** The time-of-addition experiment with artesunate was carried out in A549-hACE2 cells as described in Materials and Methods. The image of the 96-well plate is shown prior to (top image) and following (bottom image) automated counting of single SARS-CoV-2 spike glycoprotein positive cells. Unless otherwise specified, each condition was tested with 6 replicates.

Row A: Artesunate was added upon inoculation with virus (0 hours post inoculation) and removed together with the virus following the 2h infection phase.

Row B: Artesunate was added 2 h post inoculation, immediately after the 2h viral infection phase.

Row C: Artesunate was added 4 hours post inoculation.

Row D: Artesunate was added 6 hours post inoculation.

Row E and F: Infected non-treated controls (12 replicates).

Row G: Non-infected controls.

# 7. References

S1) Horváth, Z.; Horosanskaia, E.; Lee, J. W.; Lorenz, H.; Gilmore, K.; Seeberger, P. H.; Seidel-Morgenstern, A. Recovery of Artemisinin from a Complex Reaction Mixture Using Continuous Chromatography and Crystallization. *Org. Process Res. Dev.* **2015**, *19*, 624-634. [DOI:10.1021/acs.oprd.5b00048](https://doi.org/10.1021/acs.oprd.5b00048).

S2) Bussmann, B. M.; Reiche, S.; Jacob, L. H.; Braun, J. M.; Jassoy, C. Antigenic and cellular localization analysis of the severe acute respiratory syndrome coronavirus nucleocapsid protein using monoclonal antibodies. *Virus Res.* **2006**, *122*, 119-126. DOI: 10.1016/j.virusres.2006.07.005.

S3) von Einem, J.; Schumacher, D.; O’Callaghan, D. J.; Osterrieder, N. The α-TIF (VP16) homologue (ETIF) of equine herpesvirus 1 is essential for secondary envelopment and virus egress. *J. Virol.* **2006**, 2609-2620. DOI: 10.1128/JVI.80.6.2609–2620.2006.
